# A columnar liquid crystalline self-assembly of a donor–acceptor TADF emitter design for solution-processed OLEDs

**DOI:** 10.1039/d6sc02453j

**Published:** 2026-05-15

**Authors:** Joydip De, Yuka Yasuda, Mikihito Takenaka, Amy Drysdale-Dykes, Hironori Kaji, Eli Zysman-Colman

**Affiliations:** a Organic Semiconductor Centre, EaStCHEM School of Chemistry, University of St Andrews St Andrews KY16 9ST UK eli.zysman-colman@st-andrews.ac.uk; b Institute for Chemical Research, Kyoto University Uji Kyoto 611-0011 Japan kaji@scl.kyoto-u.ac.jp

## Abstract

The self-assembly of π-conjugated molecules into supramolecular columnar structures has become an effective strategy for the creation of soft, durable, and adaptable materials with immense potential for application in organic optoelectronic devices. In this regard, the columnar organization of discotic liquid crystals (DLCs) is well-studied in terms of a quasi-1D charge transport medium, which can be exploited across a range of organic electronic device applications. There are relatively few examples of room temperature columnar DLCs emitting thermally activated delayed fluorescent (TADF). Herein, we demonstrate a molecular design strategy to deliver a material that simultaneously shows bright and efficient TADF and self-organizes into columnar DLCs at room temperature. The compound TCzTRZ-DLC contains three dendrimeric carbazole-based donors with mesogenic units decorating a central 1,3,5-triphenyltriazine acceptor. Notably, the system exhibited a desired homeotropic alignment resulting in preferential aromatic π-stacking among disc-like molecules, which is beneficial to boost the light out-coupling efficiency in solution-processed organic light-emitting diodes (OLEDs). The resulting green-emitting SP-OLEDs emitted at *λ*_EL_ of 488 nm and showed a maximum external quantum efficiency, EQE_max_ of 15.5%. This represents a significant improvement in OLED efficiency compared to other solution-processed devices using TADF emitters bearing mesogenic groups.

## Abstract 2

The self-assembly of π-conjugated molecules into supramolecular columnar structures has become an effective strategy for the creation of soft, durable, and adaptable materials with immense potential for application in organic optoelectronic devices.

## Graphical abstract

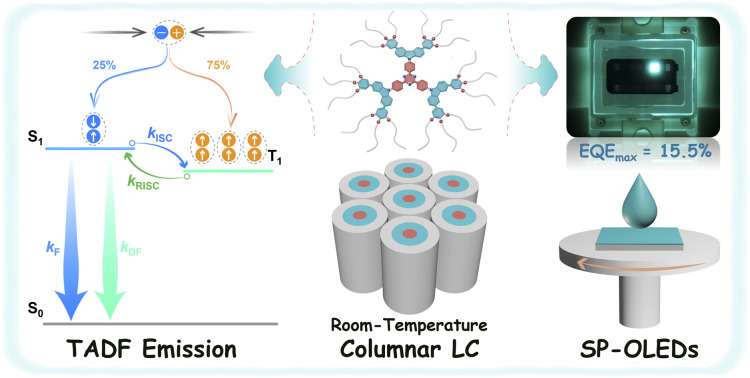

## Introduction

The supramolecular self-assembly of organic semiconductor molecules is a powerful strategy for the construction of functional soft materials with promising applications in the fields of photonics and optoelectronics.^1–6^ In this context, liquid crystals (LCs) represent a distinctive class of soft matter that combines the orientational order of crystalline solids with the fluidity of isotropic liquids, enabling dynamic structural responsiveness from molecular to macroscopic scales.^7^

Emissive LCs have garnered particular interest as the supramolecular organization^8,9^ can facilitate anisotropic charge transport, lowering drive voltages and enhancing the efficiency of solution-processed organic light-emitting diodes (SP-OLEDs).^10–13^ The major limitation of fluorescent LCs as emitters for SP-OLEDs is their poor exciton utilization efficiency, which translates into a theoretical maximum internal quantum efficiency (IQE_max_) of only 25%. An initial approach to address this design limitation was introduced by Bruce and co-workers,^14^ who developed a series of phosphorescent LCs based on metallomesogens, some of which were subsequently implemented in OLEDs and the most efficient of which showed a maximum external quantum efficiency (EQE_max_) of 7.1%,^15^ where the emissive layer consisted of 5 wt% of a mesogenic gold(iii) complex (compound 10c) bearing modified pincer and acetylide ligands as the emitter in a PVK : OXD-7 (7 : 3) blended host.^15^ This efficiency is far below the state-of-the-art for SP-OLEDs.^16,17^

**Fig. 1:**
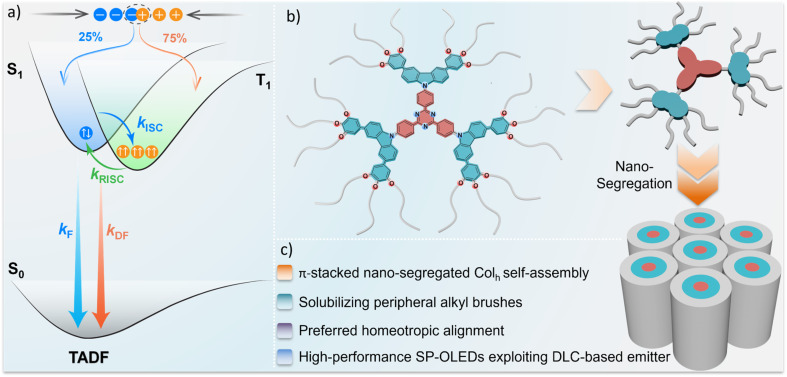
Simplified state diagram showing (a) exciton recombination process following electrical excitation; (b) molecular structure of TCzTRZ-DLC containing dodecyl mesogenic groups along with an illustrated overview of their self-assembly, which produces a columnar hexagonal mesophase; and (c) a summary of key outcomes of this study.

An alternative class of emissive LCs that can be used in devices capable of reaching up to 100% IQE_max_ is thermally activated delayed fluorescent (TADF) LCs. TADF emitters typically adopt a highly twisted donor–acceptor (D–A) geometry, which reduces the exchange integral between the frontier molecular orbitals and consequently narrows the singlet–triplet energy gap, Δ*E*_ST_ sufficiently to enable a thermally promoted endothermic upconversion of triplet excitons into singlets (Fig. 1).^18^ To date, there have been a small number of reports of D–A TADF LC emitters. Their mesophase behavior, key photophysical properties, and associated SP-OLED device metrics (including emissive layer composition), are summarized in Fig. S1. Bruce and co-workers reported a family of terephthalonitrile derivatives, 2,5-di(*N*,*N*′-carbazolyl)terephthalonitrile (1a) and 2,3,5,6/2,4,5,6-tetra(*N*,*N*′-carbazolyl)terephthalonitrile (1b/1c), that are functionalized with 3,4-didodecyloxyphenyl mesogenic groups at the 3- and 6- positions of the carbazole donor units (Fig. S1).^19,20^ These materials adopt a columnar hexagonal (Col_h_) mesophase with phase transitions: 1a, Col_h_ 181 °C Iso (isotropic); 1b, Col_h_ 191 °C Iso; and 1c, glassy (g) 35 °C Col_h_ 76 °C Iso. When compound 1c was used as the emitter (1 wt%) in a PVK : OXD-7 (7 : 3) blended host, the corresponding SP-OLED showed an EQE_max_ of 3.0%; notably, in this report, the functional OLED is divorced from the LC properties of the neat film.^20^ Subsequently, Wang and co-workers reported *p*-DPSAc-LC,^21^ where the diphenylsulfone serves as the acceptor, acridine as the donor, and functionalized biphenyl mesogenic groups. The neat film of *p*-DPSAc-LC adopts a Smectic A mesophase between 88–100 °C. As a doped film in mCP (doping concentration not provided), *p*-DPSAc-LC emits at *λ*_PL_ of 492 nm, has a delayed fluorescence lifetime (*τ*_d_) of 48.01 µs and a photoluminescence quantum yield (*Φ*_PL_) of 66.5%, while the neat film emits at *λ*_PL_ of 516 nm, with *τ*_d_ of 1.95 µs and (*Φ*_PL_) of 54.7%. The corresponding SP-OLEDs with an EML consisting of 20 wt% *p*-DPSAc-LC doped in mCP showed an EQE_max_ of 14.9%, emitting at *λ*_EL_ of 500 nm [CIE coordinate of (0.21, 0.44)].^21^ The same group developed a chiral functionalized binaphthol derivative *R*/*S*-4,^22^ which forms a Smectic A* mesophase as a neat film and emits at *λ*_PL_ of 523 nm. As a 5 wt% doped film in mCP, *R*/*S*-4 emits at *λ*_PL_ of 521 nm and has a Δ*E*_ST_ of 0.05 eV, a *τ*_d_ of 4.4 µs, *Φ*_PL_ of 51%; neat film photophysics were not provided. The SP-OLEDs with 5 wt% *R*-4 in mCP showed an EQE_max_ of 10.2% (Fig. S1).

Recently, our group reported the first example of a LC containing a multiresonant TADF (MR-TADF) emissive core, DiKTa-LC.^23^DiKTa-LC exhibited a nematic discotic (N_D_) mesophase^23^ and a preferential horizontal orientation of its transition dipole moment, characterized by an anisotropy factor (*a*) of 0.28, which is preserved in doped poly(vinylcarbazole) (PVK) films. This horizontal orientation was leveraged to enhance the light outcoupling from the SP-OLEDs using DiKTa-LC in the emissive layer, which showed an EQE_max_ of 13.6% at an *λ*_EL_ of 492 nm. Subsequent collaborative work with Knöller *et al.* on boron-based MR-TADF LCs showed that these materials assembled into a Col_h_ mesophase that is stable between 22–144 °C. The LC emits at *λ*_PL_ of 544 nm and has *Φ*_PL_ = 39% in the neat film; its Δ*E*_ST_ is 0.25 eV in toluene glass.^24^ Another boron-containing MR-TADF Col_h_ LC was exploited as a host of a small molecule MR-TADF emitter BCzBN. This host-guest strategy resulted in efficient Förster resonance energy transfer (FRET) from host to guest, enabling narrowband emission from the latter.^25^ Among these examples, only DiKTa-LC,^23^ had been employed as an emitter in SP-OLED devices, where the emissive layer consisted of 10 wt% DiKTa-LC doped in PVK. However, in all reported examples, the emissive layers employed in devices were not liquid-crystalline, and thus the potential advantages of employing emissive mesogens remain largely unrealized.

Of the accessible mesophases, discotic columnar liquid crystalline (DLC) mesophases are of particular interest as these can self-assemble to enable efficient charge transport along the columnar director.^26–29^ Fluorescent compounds capable of forming columnar DLC phases have also been investigated as emitters in OLEDs, for instance, alkylated triazole-substituted perylene tetraesters, which when doped at 5 wt% in CBP host, yielded devices with an EQE_max_ of 6.5%.^30^ To date, only one report exists of a D–A TADF columnar DLC used in an OLED,^20^ which when used in an emissive layer consisting of 1 wt% emitter doped in a 7 : 3 blended host of PVK : OXD-7, the device with 1c showed a low EQE_max_ of 3.0%. The low EQE_max_ was attributed by the authors to be due to the poor carrier mobility resulting from the insulating mesogenic chains.^19,20^

**Scheme 1:**
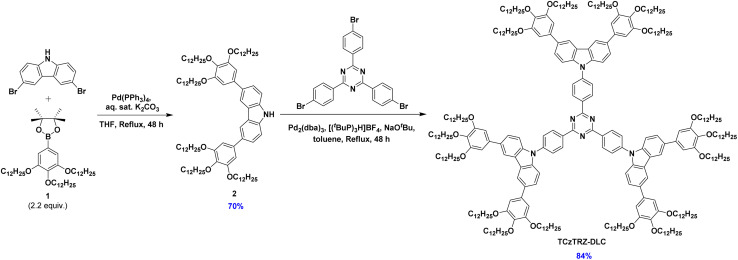
Synthesis of TCzTRZ-DLC.

Here we report a D–A TADF DLC compound TCzTRZ-DLC (Scheme 1) that contains a central 2,4,6-triphenyl-1,3,5-triazine acceptor that is decorated with three 3,6-bis(3,4,5-tris(dodecyloxy)phenyl)-9*H*-carbazole donors, each of which contains two mesogenic units. The molecular design builds upon previously reported deep blue fluorescent *C*_3_-symmetric tricarbazolyl triazine derivatives.^31,32^ We hypothesized that by incorporating electron-donating mesogenic substituents at the 3- and 6-positions of the carbazole donors, the corresponding compound TCzTRZ-DLC would show red-shifted TADF and self-assemble into a stable columnar mesophase. Indeed, spin-coated neat and doped films in bis[2-(diphenylphosphino)phenyl]ether oxide (DPEPO) reveals comparable TADF photophysics. The neat film of TCzTRZ-DLC emits at *λ*_PL_ of 498 nm, has a *Φ*_PL_ of 55% and a rather short *τ*_d_ of 2.25 µs, while in the 10 wt% doped film in DPEPO the emission is slightly blue-shifted at *λ*_PL_ of 490 nm, the *Φ*_PL_ increases to 81% while the *τ*_d_ is comparable at 2.55 µs. Neat films are liquid crystalline over a wide temperature window from room-temperature to 145 °C. SP-OLEDs with an EML consisting of 10 wt% TCzTRZ-DLC doped in mCP showed an EQE_max_ of 9.3% and emitted at *λ*_EL_ of 492 nm, while the introduction of OXD-7 as an electron-transporting co-host (10 wt% TCzTRZ-DLC:30 wt% OXD-7:60 wt% mCP) resulted in devices with an EQE_max_ of 15.5%, emitting at *λ*_EL_ of 488 nm [CIE coordinates of (0.19, 0.37)]. This work demonstrates a step-change in performance for columnar SP-OLEDs employing doped emissive layers containing DLC TADF emitters and reveals that the photophysics of the emissive core can largely be conserved as a neat film and that there is no significant aggregation-caused quenching (Fig. 1).

## Results and discussion

### Synthesis

The synthesis of TCzTRZ-DLC is shown in Scheme 1. Key intermediate 2 was obtained following a Suzuki–Miyaura cross-coupling reaction between 3,6-dibromo-9*H*-carbazole and two equivalents of 4,4,5,5-tetramethyl-2-(3,4,5-tris(dodecyloxy)phenyl)-1,3,2-dioxaborolane (1) in 70% yield; compound 1 was accessed in 3 steps following a literature procedure^33^ (Scheme S1). TCzTRZ-DLC was obtained following a three-fold Buchwald–Hartwig amination between 2 and 2,4,6-tris(4-bromophenyl)-1,3,5-triazine in 84% yield. TCzTRZ-DLC was purified by silica gel chromatography followed by gel permeation chromatography. The identity and purity of the intermediates and targeted emitter were characterized by a combination of ^1^H and ^13^C nuclear magnetic resonance (NMR) spectroscopy, high-resolution mass spectrometry (HRMS), melting point determination, elemental analysis (EA), and high-performance liquid chromatography (HPLC) (Fig. S2–S9). The thermal stability of TCzTRZ-DLC was evaluated by thermogravimetric analysis (TGA) under a nitrogen atmosphere. TCzTRZ-DLC has a decomposition temperature (*T*_d_, corresponding to the 5% weight loss) of 366 °C (Fig. S10), whereas the decomposition of 2,4,6-tris(4-(3,6-di-*tert*-butyl-9*H*-carbazol-9-yl)phenyl)-1,3,5-triazine (TPT-DB) was reported to start at 184 °C.^32^

### Mesogenic properties

**Fig. 2:**
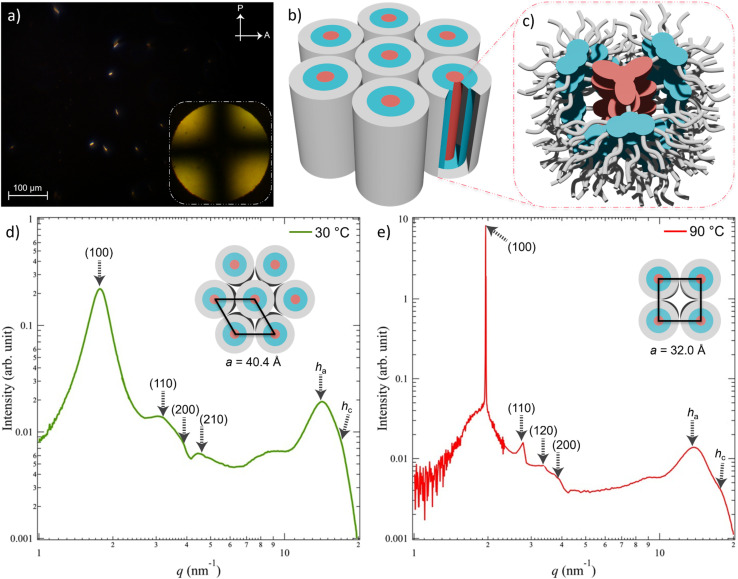
POM image of TCzTRZ-DLC placed between a glass slide and a coverslip under crossed polarization angle of 90° at 30 °C (inset in (a) shows the conoscopic image). Arrows indicate the directions of A: analyser; P: polarizer. (b and c) Schematic illustration of the homeotropically aligned Col_h_ self-assembly of TCzTRZ-DLC molecules. X-ray scattering pattern of TCzTRZ-DLC at (d) 30 °C and (e) 90 °C. Insets of (d) and (e) show the schematic illustration of the 2D-lattice of Col_h_ and Col_sq_ mesophase, respectively.

We first investigated the mesogenic properties of TCzTRZ-DLC using a combination of differential scanning calorimetry (DSC), polarized optical microscopy (POM), and small and wide-angle X-ray scattering (SAXS/WAXS). The DSC scan (Fig. S11 and Table S1) revealed two transitions, one at 65.9 °C (Δ*H* = 8.84 kJ mol^−1^) and a second at 143.1 °C (Δ*H* = 1.32 kJ mol^−1^). The first transition is attributed to columnar hexagonal (Col_h_) to columnar square (Col_sq_) mesophase transformation, whereas the second transition corresponds to the Col_sq_ mesophase to isotropic transition, as evidenced from SAXS/WAXS analysis (*vide infra*). The POM image in Fig. 2a of TCzTRZ-DLC shows mostly dark areas and contains minor birefringence having rectilinear defects in its texture, indicating a columnar type mesophase. The dark regions were further examined by conoscopy to interrogate the type of oriented domains that cause this dark appearance. Fig. 2a (inset) reveals that the split isogyres cross exactly at the center, which is governed by the emergence of a single optical axis over the monodomain, confirming the homeotropic alignment of the sample. Fig. 2b represents the plausible schematic illustration of the assembly of TCzTRZ-DLC molecules in their homeotropically oriented state over a micrometer range of thickness.

Small and wide-angle X-ray scattering (SAXS/WAXS) measurements at 30 °C and 90 °C provide insight as to the supramolecular packing arrangement of TCzTRZ-DLC. Fig. 2c shows the SAXS/WAXS profile of TCzTRZ-DLC at 30 °C. In the small-angle (1 < *q* < 5 nm^−1^) regime, it reflects one strong and four weak peaks with a peak ratio of 
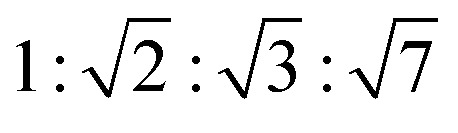
 that have been assigned to the (100), (110), (200) and (210) planes of the hexagonal lattice having a lattice parameter, *a* = 40.4 Å (Table S2). In the wide-angle (at higher *q*) region, the measurement exhibits a broad halo at a *d*-spacing of 4.5 Å, which belongs to the inter-chain correlation of alkyl-chains, and another peak at the *d*-spacing of 3.5 Å, which arises due to the π–π interaction between two adjacent discs, clearly suggesting a columnar π stacking of the molecules. Therefore, at 30 °C, the mesophase of the self-assembled TCzTRZ-DLC is columnar hexagonal (Col_h_) in nature. At higher temperature (90 °C), TCzTRZ-DLC scatters one sharp and three weak signals with a peak ratio of 
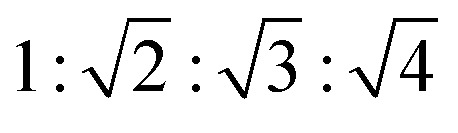
 that have been assigned to the (100), (110), (120) and (200) planes of the square lattice having a lattice parameter, *a* = 32.0 Å (Fig. 2d and Table S2). Additionally, there are two similar (as seen at 30 °C) signals indicating inter-chain correlation of alkyl brushes and π–π interaction of the discotic cores, confirming that at 90 °C TCzTRZ-DLC self-assembles into a columnar square (Col_sq_) mesophase. At 90 °C, the POM texture of TCzTRZ-DLC, did not show significant differences (Fig. S12), which likely reflects the strong tendency of these columnar phases to orient perpendicular to the substrate despite the change in lattice symmetry.

**Fig. 3:**
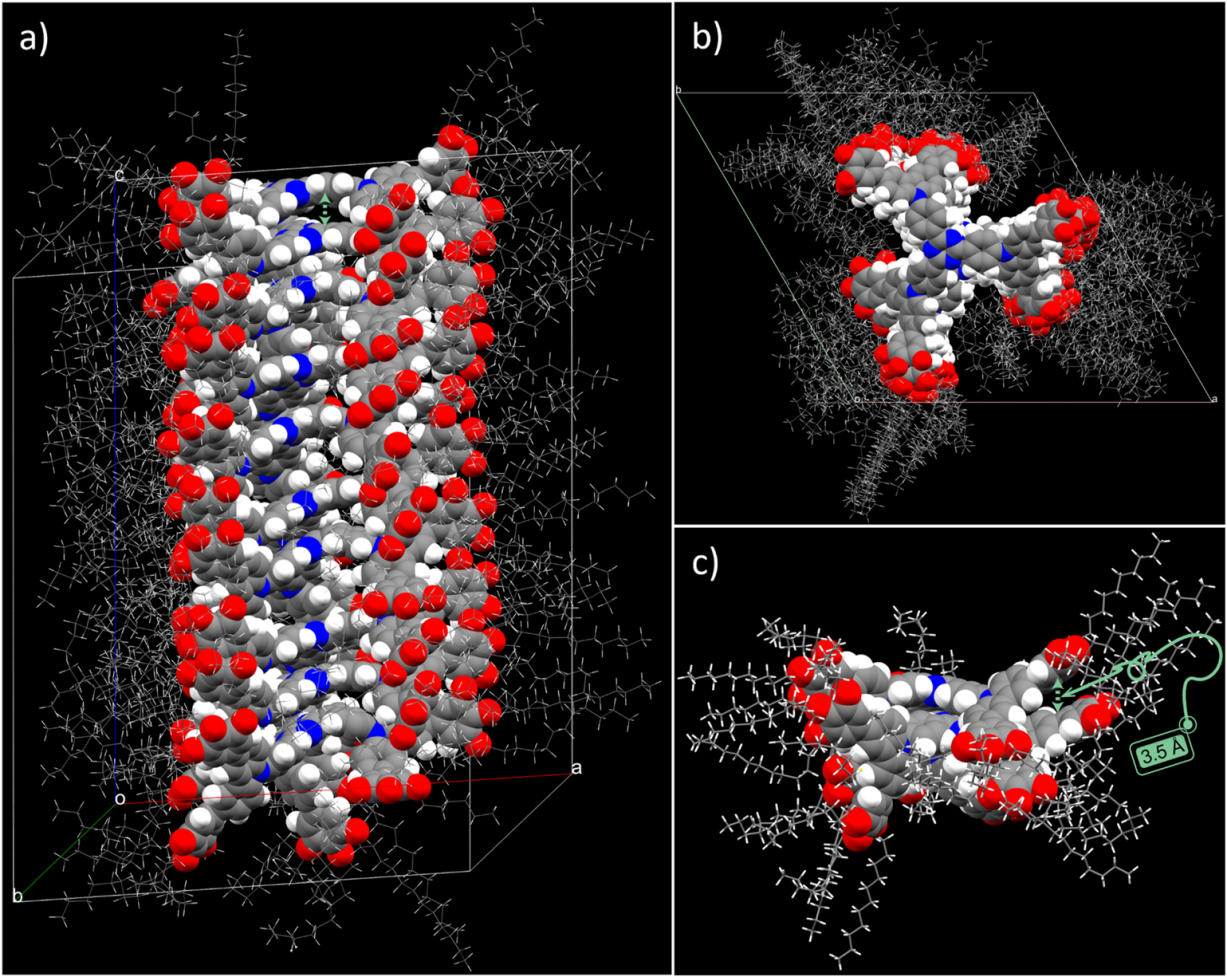
Model of the self-assembly of TCzTRZ-DLC in Col_h_ mesophase obtained from geometry optimization using the force field COMPASS (Forcite Plus module). Stacking of ten molecules along the columnar stack in the hexagonal lattice (a) side view, (b) top view. (c) Enlarged side view of the molecular dimer highlighting the axial translation of 3.5 Å corresponding to the π–π stacking distance. In (a and b) molecular structures represented as space-filling model for the central aromatic motifs, while the peripheral aliphatic brushes as wireframe model; in (c), aliphatic segments shown as ellipsoid model, aromatic parts as space-filling model (atom color code: C, gray; N, blue; O, red; H, white).

Based on the structural parameters obtained from the SAXS/WAXS measurements, the packing structure of the ambient-temperature mesophase (Col_h_) of TCzTRZ-DLC was modelled using the BIOVIA Materials Studio 2017 R2 program package to gain molecular-level insight into what governs the self-assembly. The packing in the Col_h_ state of TCzTRZ-DLC was modelled using the force-field Forcite Plus (COMPASS) module employing an Ewald summation method. The optimized superstructure exhibited a negative non-bonding interaction energy (−544 kcal mol^−1^), indicating a thermodynamically favourable assembly. Fig. 3a illustrates the geometry-optimized structure where the TCzTRZ-DLC molecules organize in a nano-segregated fashion along the columnar axis through synergistic π–π-stacking and van der Waals interactions forming a well-defined column in the hexagonal lattice, which is clearly seen from the top view in Fig. 3b and c shows a π–π-stacking separation of 3.5 Å among adjacent discs of TCzTRZ-DLC. This self-assembled structure indicates a uniaxial homeotropic alignment of the hexagonal lattice, consistent with a circular cross-section of the columnar core formed by the disk-like TCzTRZ-DLC chromophores. Such an organization arises upon cooling from the isotropic melt between the glass substrate and cover slip, as confirmed by the conoscopic pattern (Fig. 2a, inset).

### Theoretical calculations

**Fig. 4:**
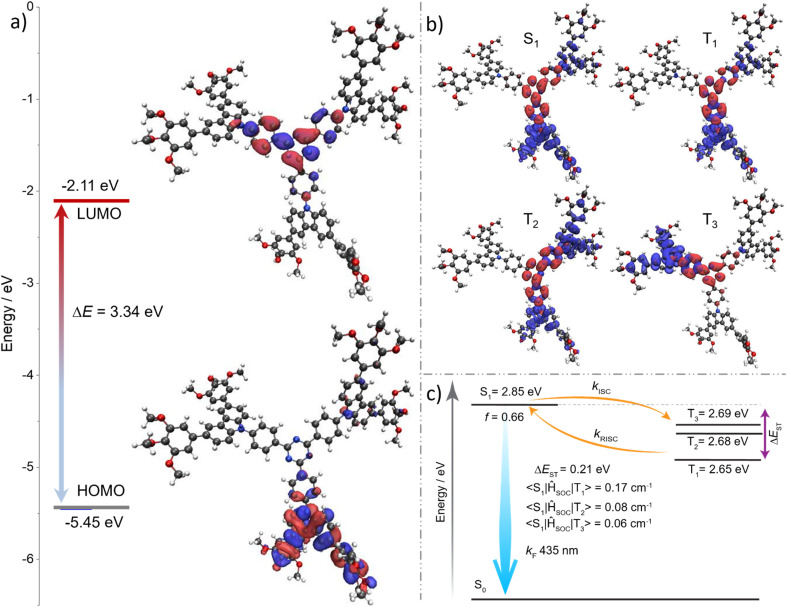
(a) Electronic distributions of the frontier molecular orbitals (isovalue: 0.02) of TCzTRZ-OMe, calculated in the gas phase at the PBE0/6-31G(d,p) level. (b) The natural transition orbitals (NTOs) (isovalue: 0.02) for S_1_, T_1_, T_2_ and T_3_ states, along with (c) the energy levels of the excited states calculated at the TDA-DFT-PBE0/6-31G(d,p) level in the gas phase based on the ground-state optimized geometry, and spin–orbit coupling matrix element (SOCME) calculated at the optimized T_1_ geometry in the gas phase at the PBE0/6-31G(d,p) level for TCzTRZ-OMe. The red color represents an area of increasing electron density, and the blue colour represents a decreased electron density.

The chemical structure of TCzTRZ-DLC is inspired by the structure of the previously reported fluorophore 2,4,6-tris(4-(9*H*-carbazol-9-yl)phenyl)-1,3,5-triazine (TPT-Cz),^31,32^ consisting of three carbazole (Cz) donors decorating a triphenyltriazine (TPT) acceptor (here, we refer to the triphenyltriazine unit as TRZ), which emits at *λ*_PL_ of 432 nm in toluene.^32^ Calculations of this compound conducted at the PBE0/6-31G(d,p) level of theory in the gas phase predict a large Δ*E*_ST_ of 0.34 eV (S_1_ = 3.14 eV and T_1_ = 2.80 eV), which is a bit large to enable TADF. To understand the impact on the optoelectronic properties resulting from the addition of the mesogenic aryl groups onto the carbazole donor, we modelled both TCzTRZ-DLC as well as a reference compound in which the mesogenic moieties were replaced by methoxy groups, TCzTRZ-OMe. Calculations were carried out at the PBE0/6-31G(d,p) level of theory in the gas phase.^34,35^ We have previously shown this level of theory to be sufficient to accurately model the optoelectronic properties of D–A TADF compounds.^36^ Unsurprisingly, both TCzTRZ-OMe and TCzTRZ-DLC possess similar electron density distributions of the highest occupied and lowest unoccupied molecular orbitals, HOMO and LUMO (Fig. 4a and S13). For both TCzTRZ-OMe and TCzTRZ-DLC, the HOMO is mainly located on one of the three substituted-carbazole units, whereas the LUMO is located on the central TRZ moiety. The HOMO and LUMO energies of TCzTRZ-OMe are −5.45 and −2.11 eV, respectively. Compared to TCzTRZ-OMe, the HOMO level of TCzTRZ-DLC is stabilized by 0.07 eV at −5.52 eV, while the LUMO level is stabilized by 0.02 eV at −2.13 eV (Fig. S13). Consequently, the HOMO/LUMO energy gap (Δ*E*_g_) of TCzTRZ-DLC is slightly widened to ∼3.39 eV (Fig. S13) compared with 3.34 eV for TCzTRZ-OMe (Fig. 4a).

Given that there is negligible demonstrated impact from the presence of the mesogenic units on the HOMO/LUMO levels, we thus only modelled the excited states of the reference, TCzTRZ-OMe. The calculated energies of the S_1_ and T_1_ states of TCzTRZ-OMe are 2.85 and 2.65 eV, respectively, while the Δ*E*_ST_ is 0.21 eV. Thus, the incorporation of the trialkoxy-substituted aryl groups onto the donor unit significantly strengthens the donor and this leads to the smaller Δ*E*_ST_ value compared to the unsubstituted TPT-Cz (Δ*E*_ST_ = 0.34 eV); notably, there is also an increased density of low-lying triplet states (Fig. 4b and c), both of which should produce a faster reverse intersystem crossing (RISC) and manifest in TADF.^37–39^ Both S_1_ and T_1_ have similar electron density distributions yet non-negligible spin–orbit coupling. The high oscillator strength (*f* = 0.66) for the S_0_ → S_1_ transition, coupled with the moderately large Δ*E*_ST_, suggests that the emissive charge-transfer state contains an admixture of locally excited character. The transition dipole moment (TDM) vector of TCzTRZ-OMe for the S_0_ → S_1_ transition is oriented only 0.06° off the horizontal relative to the molecular plane (*X*/*Y* plane in Fig. S13b). This indicates the potential for the TDMs of molecules of TCzTRZ-DLC to co-align in a columnar mesophase, oriented horizontally to the substrate. Such an alignment would contribute to enhancing the light out-coupling in the OLED.

### Electrochemical studies

The electrochemical properties of TCzTRZ-DLC were measured by cyclic voltammetry (CV) and differential pulse voltammetry (DPV) in deaerated dichloromethane (DCM) with 0.1 M [^*n*^Bu_4_N]PF_6_ as the supporting electrolyte. The electrochemical data reported *versus* SCE are summarized in Table S3. The CV trace (Fig. S14) shows irreversible oxidation and reduction waves. The oxidation/reduction potentials (*E*_ox_/*E*_red_) of TCzTRZ-DLC extracted from the peaks of the DPV^40^ are 1.02 and −1.72 V *vs.* SCE, respectively, corresponding to an electrochemical gap (Δ*E*) of 2.74 V. Based on these potentials, the HOMO and LUMO energy levels were estimated to be −5.36 and −2.62 eV, respectively, which qualitatively align with the DFT calculated values (Fig. S13). The estimated values of *E*_HOMO_ and *E*_LUMO_ are destabilised by 0.79 and 0.38 eV, respectively, compared to the peripherally unsubstituted analogue TPT-Cz (*E*_HOMO_ = −6.15 eV and *E*_LUMO_ = −3.00 eV).^31^ This difference likely reflects the influence of the electron-rich mesogenic substituents together with differences in the methodology used to estimate orbital energies (UPS and optical gap measurements) reported in the literature.

### Photophysical studies

**Fig. 5:**
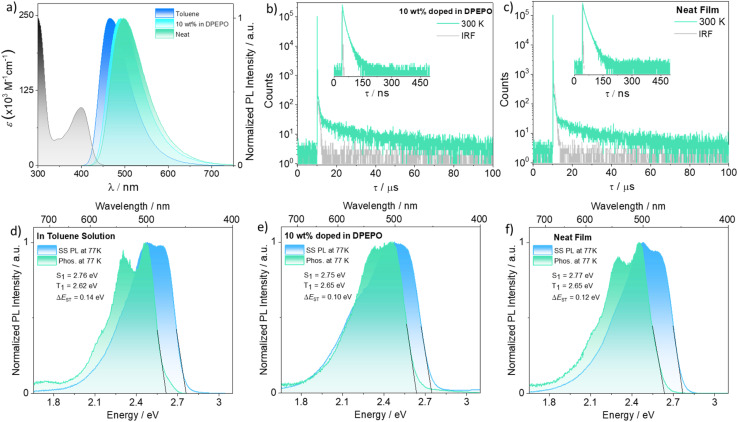
(a) Absorption and steady-state PL spectra (SS PL) in toluene solution and thin film (10 wt% in DPEPO and neat) of TCzTRZ-DLC at room temperature (excitation wavelength, *λ*_exc_ = 380 nm). Temperature-dependent time-resolved PL (TR PL) decay of TCzTRZ-DLC in (b) DPEPO at 10 wt% and (c) neat film, *λ*_exc_ = 379 nm (inset shows the prompt PL decay at 300 K). SS PL and phosphorescence spectra (1–10 ms) obtained at 77 K in (d) toluene glass (e) as 10 wt% doped film in DPEPO and (f) as neat film for TCzTRZ-DLC (*λ*_exc_ = 380 nm).

The photophysical studies of TCzTRZ-DLC in toluene are shown in Fig. 5, and the photophysical properties are summarized in Table 1. The absorption spectrum of TCzTRZ-DLC has three dominant bands (Fig. 5a). The strong band at 298 nm (molar extinction coefficient, *ε* = 2.48 × 10^5^ M^−1^ cm^−1^) is assigned to π–π* transitions of the carbazole units.^32,41^ There are additionally two other resolved bands, one at 400 nm (*ε* = 9.6 × 10^4^ M^−1^ cm^−1^) and another at 346 nm (*ε* = 4.4 × 10^4^ M^−1^ cm^−1^), which are assigned as intramolecular charge transfer (ICT) states.

**Table 1:** Photophysical data of TCzTRZ-DLC in a solution of toluene and in 10 wt% doped films in DPEPO also in the neat film

Compound	Medium	*λ*_PL_ c/nm	FWHM^d^/nm	*E*_S_1__ e/eV	*E*_T_1__ e/eV	Δ*E*_ST_^g^/eV	*Φ*_PL_ h/%	*τ*_p_ i/ns	*τ*_d_ i/µs	*k*_ISC_ j/10^7^ s^−1^	*k*_RISC_ j/10^5^ s^−1^	*k*_s_r_ j/10^7^ s^−1^	*k*_s_nr_ j/10^7^ s^−1^
TCzTRZ-DLC	PhMe a	464	74	2.76	2.62	0.14^f^	64 (52)	6.70	—	—	—	—	—
	Neat film b	498	91	2.77	2.65	0.12	55 (47)	9.15	2.25	3.65 ± 2.05	3.46 ± 1.85	5.03	4.11
	10 wt% in DPEPO b	490	85	2.75	2.65	0.10	81 (59)	6.63	2.55	9.15 ± 5.05	3.47 ± 1.91	0.9	10.1

a In Toluene solutions (10^−6^ M).

b Spin-coated thin films consisting of 10 wt% emitter in DPEPO under vacuum. *λ*_exc_ = 380 nm.

c SS PL peak maximum at 300 K. *λ*_exc_ = 380 nm.

d Full-width at half maximum.

e S_1_ and T_1_ energies extracted from the onsets of the respective prompt fluorescence (delay: 1 ns; gate time: 100 ns) and phosphorescence spectra (delay: 1 ms; gate time: 10 ms) at 77 K. *λ*_exc_ = 380 nm.

f Solution samples for Δ*E*_ST_ measurements were prepared in Toluene (10^−6^ M).

g Δ*E*_ST_ = *E*(S_1_) − *E*(T_1_).

h Relative *Φ*_PL_ in PhMe were measured using the relative method with quinine sulfate as the reference (*Φ*_r_ = 54.6% in 1 N H_2_SO_4_),^48^ while absolute *Φ*_PL_ of the thin films were measured using an integrating sphere under nitrogen (values in parenthesis represents under air).

i Prompt and delayed PL lifetimes were measured using TCSPC and MCS, respectively. *λ*_exc_ = 375 nm.

j ISC and RISC rate constants were calculated using the steady-state approximation method as described in ref. 46 and 47.

The steady-state PL (SS PL) spectrum of TCzTRZ-DLC in toluene is broad and unstructured, peaking at *λ*_PL_ = 464 nm (full-width at half maximum, FWHM of 74 nm). This profile is indicative of emission from a CT state (Fig. 5a). The PL spectrum progressively red-shifts in solvents of increasing polarity, corroborating the CT nature of the emission (Fig. S15). The *Φ*_PL_ in degassed toluene solution is 68%, which decreases to 52% in the presence of oxygen (see also Fig. S16), indicating the involvement of triplet states in the emission process. Time-resolved PL (TR PL) measurements in degassed toluene reveal mono-exponential decay kinetics with a PL lifetime (*τ*_PL_) of 6.7 ns (Fig. S17). Despite the observed oxygen sensitivity of the *Φ*_PL_, no delayed emission was detected. The singlet (S_1_) and triplet (T_1_) energies of TCzTRZ-DLC were extracted from the onsets of the SS PL and delayed emission spectra in frozen toluene at 77 K, and the Δ*E*_ST_ was determined from the energy difference between these two states. The S_1_ and T_1_ energies are 2.76 and 2.62 eV, respectively, and thus Δ*E*_ST_ is 0.14 eV (Fig. 5d). These experimental values align well with the DFT-computed ones (Fig. 4c). The measured S_1_ and T_1_ states of TCzTRZ-DLC are stabilised by 0.32 and 0.25 eV, respectively, relative to the methoxy-substituted analogue TPT-Cz-OMe (S_1_/T_1_ = 3.08/2.87 eV; Δ*E*_ST_ = 0.21 eV).^42^

We next investigated the photophysical properties of the spin-coated neat film of TCzTRZ-DLC. The SS PL is red-shifted to *λ*_PL_ of 498 nm, and the spectrum is broadened (FWHM of 91 nm, Fig. 5a). This reflects that there is some degree of aggregation in the neat film. The extracted S_1_ and T_1_ energies from the 77 K measurements are 2.77 and 2.65 eV, respectively, with a Δ*E*_ST_ of 0.12 eV. These values are very similar to those in toluene. Notably, the 77 K emission spectrum does not show distinct bands from an aggregate, suggesting that the contribution to the PL spectrum from aggregates is small at low temperature, consistent with previous observations in numerous TADF systems.^43,44^ The *Φ*_PL_ of the neat film under N_2_ is 54.8%, while in air it is 46.6% (Table S4). TR PL measurements reveal biexponential decay kinetics, with associated prompt and delayed PL lifetimes of *τ*_p_ of 9.15 ns [*τ*_1p_ = 5.35 ns (36%) and *τ*_2p_ = 15.10 ns (64%)] and *τ*_d_ of 2.25 µs [*τ*_1d_ = 0.71 µs (29%) and *τ*_2d_ = 17.57 µs (71%)] at 300 K (Fig. 5c, f and Table 1). Temperature-dependent TR PL measurements show that the prompt emission is insensitive to temperature, whereas the magnitude of the delayed emission intensity increases with increasing temperature (Fig. S18). Thus, the neat films show TADF behavior.

We also assessed the photophysical properties of TCzTRZ-DLC doped in three different host matrices possessing suitably high triplet energies (mCP, OXD-7, and DPEPO) and across a range of doping concentrations (Table S4). It was observed that the 10 wt% doped film of TCzTRZ-DLC in DPEPO (*E*_T_ = 2.98 eV)^45^ has the highest *Φ*_PL_ of 81.2% (under N_2_). This film emits at *λ*_PL_ = 490 nm (FWHM of 85 nm), an emission that is only slightly hypsochromically shifted compared to that of the neat film. The S_1_/T_1_ energies are 2.75/2.65 eV, and the Δ*E*_ST_ value is 0.10 eV for the 10 wt% TCzTRZ-DLC doped film in DPEPO (Fig. 5e), again very similar values to those measured in frozen toluene. In contrast, the room-temperature SS PL spectra of the neat and doped films are red-shifted and broadened relative to that in toluene solution, reflecting in part the higher polarity of the solid-state environment rather than emission from an aggregate. The TR PL decay of the 10 wt% doped film of TCzTRZ-DLC in DPEPO is biexponential, with a *τ*_p_ of 6.63 ns [*τ*_1p_ = 4.75 ns (57%) and *τ*_2p_ = 13.93 ns (43%)] and a *τ*_d_ of 2.55 µs [*τ*_1d_ = 0.76 µs (27%) and *τ*_2d_ = 19.50 µs (73%)], values that are practically identical to those of the neat film (Fig. 5b). The temperature-dependent TR PL behavior is similar to that observed for the neat film, so the 10 wt% doped film of TCzTRZ-DLC in DPEPO also is TADF. The excitonic rate constants were calculated following the approach outlined in our earlier work^46,47^ (Table 1). The intersystem crossing (ISC) rate constants (*k*_ISC_) are 3.65 × 10^7^ s^−1^ for the neat film and 9.15 × 10^7^ s^−1^ for the 10 wt% doped film in DPEPO. The corresponding RISC rate constants (*k*_RISC_) are nearly identical at 3.46 × 10^5^ s^−1^ and 3.47 × 10^5^ s^−1^, respectively. The singlet radiative (*k*_s_r_) decay rate constants are as 5.03 × 10^7^ s^−1^ for the neat film, and 0.90 × 10^7^ s^−1^ in the doped film, respectively.

### Solution-processed OLEDs

**Fig. 6:**
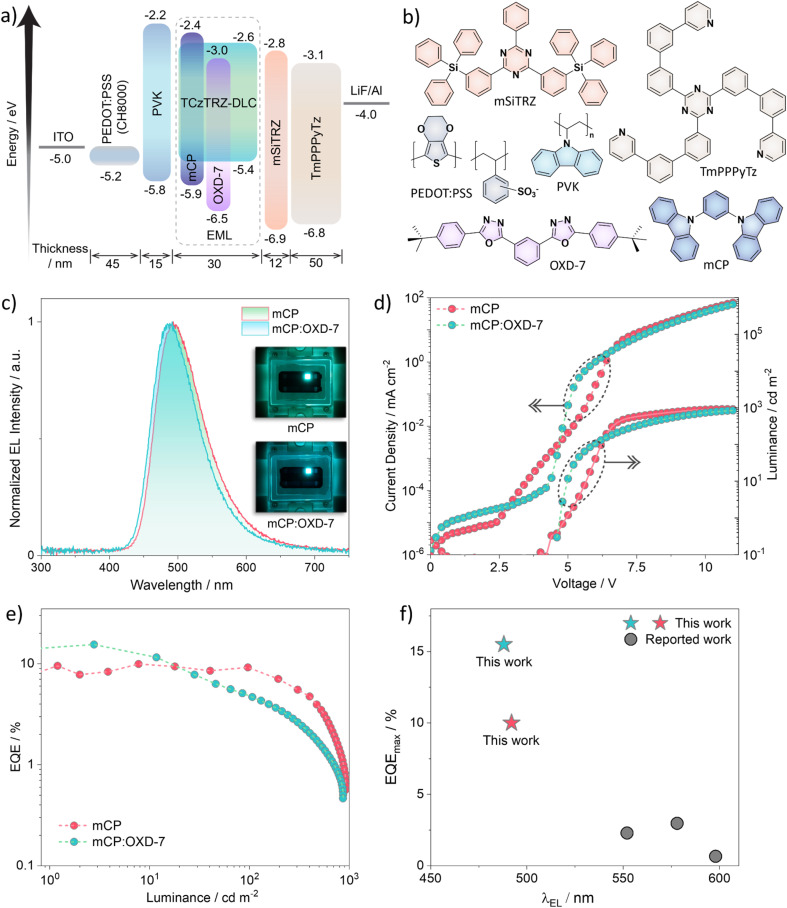
(a) Device structures with energy levels of the materials used in fabricating the SP-OLEDs, (b) chemical structures of the materials employed in the devices. (c) Electroluminescence spectra, (d) current density–voltage–luminance characteristics, and (e) EQE *versus* luminance curves of SP-OLEDs based on TCzTRZ-DLC as emitter. Insets of (c) show the images of glowing OLED devices. (f) EQE_max_*vs. λ*_EL_ comparison between the devices reported in this study and previously reported columnar LC-based TADF SP-OLEDs.

SP-OLEDs were fabricated with an EML consisting of 10 wt% TCzTRZ-DLC doped in mCP or OXD-7. The device structure is shown in Fig. 6a (materials shown in Fig. 6b) and comprised ITO (50 nm)/PEDOT:PSS (45 nm)/PVK (15 nm)/EML (30 nm)/mSiTRZ (12 nm)/TmPPPyTz (50 nm)/Liq (1 nm)/Al (100 nm). PEDOT:PSS acts as the hole injection layer, PVK served as a hole-transporting and electron-blocking layer, mSiTRZ acted as a hole/exciton-blocking layer, TmPPPyTz enables electron transport, and Liq promotes electron injection from the Al cathode. This architecture was designed to ensure exciton confinement within the EML while maintaining carrier balance through appropriate hole- and electron-transporting layers.

Given that the triplet energy of TCzTRZ-DLC is 2.65 eV, mCP (*E*_T_ ≈ 2.9 eV) was first employed as a suitable host to confine triplet excitons onto the emitter. The 10 wt% TCzTRZ-DLC:mCP film has a *Φ*_PL_ of 71.4% under N_2_. The corresponding device emitted at *λ*_EL_ = 492 nm (FWHM of 80 nm, Fig. 6c), closely matching the PL profile. The EQE_max_ reached 9.3% (Fig. 6d and Table 2), indicating incomplete exciton utilization given that the theoretical maximum value is ∼14%. The current efficiency (CE) *vs.* luminance and power efficiency (PE) *vs.* luminance characteristics (Fig. S19 and S20) reveal moderate efficiency roll-off at higher luminance, suggesting an imbalance in carrier transport within the emissive layer. The predominantly hole-transporting nature of mCP, combined with the directional π–π stacking (*d* = 3.5 Å) inherent to the columnar organization of TCzTRZ-DLC, likely favors hole transport over electron mobility, leading to poor exciton utilization.

**Table 2:** Device performance of SP-OLEDs

Emissive layer (EML)	*V*_on_ a/V	EQE_max_/EQE_100_/EQE_1000_^b^/%	*λ*_EL_ c/nm	FWHM^d^/nm	CIE^e^ (*x*, *y*)
10 wt% TCzTRZ-DLC:mCP	5.2	9.3/9.2/0.62	492	80	(0.21, 0.42)
10 wt% TCzTRZ-DLC:30 wt% OXD-7:60 wt% mCP	4.8	15.5/4.7/0.50	488	77	(0.19, 0.37)

a Turn-on voltage at 1 cd m^−2^.

b Maximum the external quantum efficiency (EQE) and EQE at 100 and 1000 cd m^−2^.

c Electroluminescence spectra maximum.

d full-width at half maximum, and.

e Commission Internationale de l’Éclairage (CIE) at current densities at 1 A m^−2^.

To improve the charge carrier balance, OXD-7 was introduced as an electron-transporting co-host (10 wt% TCzTRZ-DLC:30 wt% OXD-7:60 wt% mCP). This change in host composition produced a higher device EQE_max_ at 15.5% (Fig. 6d and Table 2). The emission red-shifted slightly to 488 nm (FWHM of 78 nm, Fig. 6c), consistent with the increased polarity of the mixed host matrix and the CT nature of the emissive state.

Despite the higher EQE_max_, the co-host device exhibited a more severe efficiency roll-off relative to the device using mCP as the host (Table 2). This behavior can be rationalized by the deeper LUMO of OXD-7 relative to TCzTRZ-DLC, which promotes partial electron trapping and increases local carrier density.

The device performance is a function of the synergistic interplay between the electronic structure of the emitter in the host and supramolecular columnar organization. Importantly, when benchmarked against previously reported columnar solution-processed OLEDs using TADF emitters containing mesogenic groups (Fig. 6f and Table S5), the totality of which exhibited EQE_max_ values in the range of 0.6–3.0%, the present system delivers a substantial performance enhancement. The present study therefore, demonstrates that decorating D–A TADF emitters with mesogenic groups leads in this case to devices with much higher EQE_max_ (Fig. 6f and Table S5) compared to devices using structurally similar emitters such as TR1 and TR2.^44^

## Conclusions

In this work, we have demonstrated how the rational molecular design of organic emitters can be leveraged to develop supramolecular columnar assemblies with nano-segregated packing in the mesophase. The dendrimeric tricarbazolyl-1,3,5-triazine TCzTRZ-DLC exhibits columnar discotic liquid crystalline behavior over a broad temperature window. The molecule has a high inherent tendency to show preferential homeotropic alignment in the films, as evidenced by POM data. The incorporation of the mesogenic groups reduces the Δ*E*_ST_ and enables bright TADF emission in both doped and neat films. SP-OLEDs with TCzTRZ-DLC emitted at *λ*_EL_ of 488 nm and showed an EQE_max_ of 15.5%, which represents a significant enhancement in efficiency compared to most other SP-OLEDs using D–A TADF emitters with mesogenic groups.

## Author contributions

J. D.: conceptualization, data curation, formal analysis, funding acquisition, investigation, methodology, visualization, validation, writing – original draft, writing – review & editing. Y. Y.: data curation, formal analysis, investigation, writing – original draft, writing – review & editing. A. D. D.: data curation, formal analysis, investigation. M. T.: data curation, formal analysis, writing – original draft, writing – review & editing. H. K.: funding acquisition, methodology, project administration, resources, supervision, writing – review & editing. E. Z.-C.: conceptualization, project administration, funding acquisition, validation, visualisation, supervision, methodology, resources, writing – review & editing.

## Conflicts of interest

There are no conflicts to declare.

## Supplementary Material

SC-017-D6SC02453J-s001

## Data Availability

The research data supporting this publication can be accessed at https://doi.org/10.17630/b12abced-3700-4f5c-9123-d75a0e83e8a1. Supplementary information (SI): ^1^H NMR and ^13^C NMR spectra, HRMS, EA, HPLC of all target compounds; supplementary computational data; supplementary thermal analysis, mesophase characterization, and photophysical and device data. See DOI: https://doi.org/10.1039/d6sc02453j.
