# Supplementary material for: A columnar liquid crystalline self-assembly of a donor–acceptor TADF emitter design for solution-processed OLEDs
Source: Chem Sci. 2026 May 15;17(26):12895–906. doi: 10.1039/d6sc02453j (PMC13195308; doi:10.1039/d6sc02453j)
Supplement: SC-017-D6SC02453J-s001 [file SC-017-D6SC02453J-s001.pdf]

Supplementary Information (SI) for Chemical Science.
This journal is © The Royal Society of Chemistry 2026

Supporting Information

A Columnar Liquid Crystalline Self-Assembly of a Donor–Acceptor TADF Emitter Design for Solution-Processed OLEDs

Joydip De,[a] Yuka Yasuda,[b] Mikihito Takenaka,[b] Amy Drysdale-Dykes,[a] Hironori Kaji[b] and Eli Zysman-Colman[a]**

[a] Organic Semiconductor Centre, EaStCHEM School of Chemistry, University of St Andrews, St Andrews, UK, KY16 9ST. E-mail: eli.zysman-colman@st-andrews.ac.uk

[b] Institute for Chemical Research, Kyoto University, Uji, Kyoto, 611-0011, Japan. E-mail: kaji@scl.kyoto-u.ac.jp

Table of Contents

General Methods ---------- S2-S6

Literature Examples ---------- S7

Experimental Section ---------- S8-S16

Thermal Analysis ---------- S17-S18

Photophysical Data ---------- S18-S21

OLED Measurement Data ---------- S22-S23

References ---------- S23-S24

General Methods

General Synthetic Procedures

The starting material **1** was synthesised according to the literature procedure.[1-2] All other reagents and solvents were obtained from commercial sources and used as received. Air-sensitive reactions were performed under a nitrogen atmosphere using Schlenk techniques, no special precautions were taken to exclude air or moisture during work-up. Flash column chromatography was carried out using silica gel (Silia-P from Silicycle, 60 Å, 40-63 μm). Analytical thin-layer-chromatography (TLC) was performed with silica plates with aluminum backings (250 μm with F-254 indicator). TLC visualization was accomplished by 254/365 nm UV lamp. HPLC analysis was conducted on a Shimadzu LC-40 HPLC system. HPLC traces were performed using a Shim-pack GIST 3 μm C18 reverse phase analytical column. ^{1}H and ^{13}C NMR spectra were recorded on a Bruker Advance spectrometer (500 and 400 MHz for ^{1}H, 125 nd 100 MHz for ^{13}C) in CD_2Cl_2 and $CDCl_3$. The following abbreviations have been used for multiplicity assignments: “s” for singlet, “d” for doublet, “t” for triplet, and “m” for multiplet. ^{1}H and ^{13}C NMR spectra were referenced residual solvent peaks with respect to TMS (δ = 0 ppm). High resolution mass spectra (HRMS) were recorded on a Bruker-Daltonics autflex II (MALDI), on a Bruker-Daltonics ultrafleXtreme (HRMS-MALDI) (Bruker Daltonic GmbH, Germany). Elemental analyses were performed by the Dr. Joe Casillo at the University of Edinburgh.

Mesomorphic property measurements

The optical textures of the liquid crystalline materials were examined with a Nikon ECLIPSE LV100ND, optical polarizing microscope equipped with a Linkam LTS420 heating stage and a Linkam T95-HS system controller. Differential scanning calorimetry was performed on a Mettler Toledo DSC 1 equipped with a HSS8 ceramic sensors but no cryofill cooling system. The samples were sealed in Al crucibles and lids. All measurements were performed with a rate of 10 K/min and were evaluated with the STARe Software for Windows.

Small-angle X-ray scattering (SAXS) experiments were conducted at BL28XU of SPring-8 and using the Nano-scale Dynamic Structure Evaluation X-ray system at the Institute for Chemical Research, Kyoto University, to investigate the phase behavior of the samples. At BL28XU, the X-ray wavelength was 0.10 nm and the sample-to-detector distance was 2000 mm. A Pilatus-II detector was used. For the Kyoto University system, the X-ray wavelength and sample-to-detector distance were 0.154 nm and 1300 mm, respectively. The SAXS data were corrected for air scattering and electronic background, and the corresponding 1D profiles were obtained by circular averaging of the 2D patterns. The scattering intensity is presented as a function of the scattering vector, $q = (4\pi \sin \theta)/\lambda$, where 2θ is the scattering angle relative to the incident beam. Wide-angle X-ray scattering (WAXS) experiments were performed using the same Kyoto University system. The X-ray wavelength and sample-to-detector distance were 0.15 nm and 500 mm, respectively. The 2D WAXS data were corrected for sample absorption and for air and background scattering, and the 1D profiles were obtained by circular averaging.

Electrochemistry measurements

Cyclic Voltammetry (CV) analysis was performed on an Electrochemical Analyzer potentiostat model 620E from CH Instruments at a sweep rate of 100 mV/s. Differential pulse voltammetry (DPV) was conducted with an increment potential of 0.01 V and a pulse amplitude, width, and period of 50 mV, 0.06, and 0.5 s, respectively. Samples (**TCzTRZ-DLC**) were prepared as dichloromethane (DCM) solutions, which were degassed by sparging with DCM-saturated nitrogen gas for 5 minutes prior to measurements. All measurements were performed using 0.1 DCM solution of tetra-*n*-butylammonium hexafluorophosphate ($[^{n}Bu_4N]PF_6$]). An Ag/Ag^+ electrode was used as the reference electrode while, a glassy carbon electrode and a platinum wire were used as the working electrode and counter electrode, respectively. The redox potentials are reported relative to a saturated calomel electrode (SCE) with a ferrocenium/ferrocene (Fc/Fc^+) redox couple as the internal standard (0.46 V vs SCE).[3] The HOMO and LUMO energies were determined using the relation $E_{HOMO/LUMO} = -(E_{ox} / E_{red} + 4.8)$ eV, where E_{ox} is the anodic peak potential and E_{red} is the cathodic peak potential determined from DPV relative to Fc/Fc^+.[4]

Theoretical Calculations

Density functional theoretical (DFT) calculations and time-dependent density functional theoretical (TDDFT) calculations were performed using Gaussian 16[5] Revision D.01 software in the gas phase. The ground-state geometries were optimized employing the PBE0[6] functional with the Pople 6-31G(d,p) basis set, in the gas phase.[7] Transitions to excited singlet states and triplet states were calculated using TDDFT within the Tamm-Dancoff approximation (TDA) based on the optimized ground-state geometries.[8-9] Calculations were submitted and processed using the Digichem (version 7)[10-11] software package, which incorporates a number of publicly available software libraries, including: cclib3[12] for parsing of result files, VMD[13]/Tachyon[14] for 3D rendering, Matplotlib[15] for the plotting of graphs, Open Babel[16]/Pybel[17] for file interconversion, and PySOC[18] for the calculation of spin-orbit coupling.

Photophysical measurements

Optically dilute solutions of concentrations on the order of 10^{-5} or 10^{-6} M were prepared in spectroscopic or HPLC-grade solvents for absorption and emission analysis. Absorption spectra were recorded at room temperature on a Shimadzu UV-2600 double-beam spectrophotometer with a 1 cm quartz cuvette. Molar absorptivity determination was verified by linear regression analysis of values obtained from at least four independent solutions at varying concentrations from 8.96×10^{-6} to 2.56×10^{-6} M with absorbance ranging from 0.86 to 0.25 at 400 nm for **TCzTRZ-DLC**.

For emission studies, aerated solutions were bubbled by compressed air for 5 minutes and spectra were taken using the cuvette for absorption analysis. Degassed solutions were prepared via three freeze-pump-thaw cycles and spectra were taken using home-made Schlenk quartz cuvette. Steady-state emission, excitation spectra and time-resolved emission spectra were recorded at 298 K using an Edinburgh Instruments FS5. Samples were excited at 380 nm for steady-state PL measurements and at 379 nm for time-resolved PL measurements. Photoluminescence quantum yields, Φ_{PL}, for solutions were determined

using the optically dilute method[19] in which four sample solutions with absorbances of ca. 0.10, 0.07, 0.053 and 0.026 at 346 nm were used. The Beer-Lambert law was found to remain linear at the concentrations of the solutions. For each sample, linearity between absorption and emission intensity was verified through linear regression analysis with the Pearson regression factor (R^2) for the linear fit of the data set surpassing 0.9. Individual relative quantum yield values were calculated for each solution and the values reported represent the slope obtained from the linear fit of these results. The quantum yield of the sample, Φ_{PL}, can be determined by the equation $\Phi_{PL} = (\Phi_r * \frac{A_r}{A_s} * \frac{I_s}{I_r} * \frac{n_s^2}{n_r^2})$,[19] where A stands for the absorbance at the excitation wavelength (λ_{exc}: 346 nm), I is the integrated area under the corrected emission curve and n is the refractive index of the solvent with the subscripts "s" and "r" representing sample and reference respectively. Φ_r is the absolute quantum yield of the external reference quinine sulfate (Φ_r = 54.6% in 1 *N* H_2SO_4),[20-21] The experimental uncertainty in the emission quantum yields is conservatively estimated to be 10%, though we have found that statistically we can reproduce Φ_{PL}s to 3% relative error.

Samples for solid-state measurements were prepared by spin-coating a thin film from chloroform (Sigma-Aldrich, HPLC grade) at 2000 rpm for 60 s in an ambient environment on quartz substrates. An integrating sphere (Edinburgh Instruments FS5, SC30 module) was employed for quantum yield measurements for thin film samples. Time-resolved PL measurements of the thin films were carried out using the multi-channel scaling (MCS) and time-correlated single-photon counting (TCSPC) technique. The samples were excited at 375 nm by a picosecond pulsed diode laser (EPL-375) and were kept in a vacuum of $< 8 \times 10^{-4}$ mbar.

The singlet and triplet state energies were determined from the onset values of steady-state photoluminescence (SS PL) and time-gated (1-10 ms) phosphorescence spectra at 77 K. The singlet-triplet energy gap (ΔE_{ST}) was estimated from the difference in energy of the steady-state PL and phosphorescence spectra. The samples were excited by a xenon flashlamp emitting at 380 nm (EI FS5, SC-70).

Fitting of time-resolved PL measurements

Time-resolved PL measurements were fitted to a sum of exponentials decay model, with chi-squared (χ^2) values between 1 and 2, using the EI FS5 software. Each component of the decay is assigned a weight, (w_i), which is the contribution of the emission from each component to the total emission.

The average lifetime was then calculated using the following:

- Two exponential decay model:

$$\tau_{AVG} = \tau_1 w_1 + \tau_2 w_2$$

with weights defined as $w_1 = \frac{A1\tau_1}{A1\tau_1 + A2\tau_2}$ and $w_2 = \frac{A2\tau_2}{A1\tau_1 + A2\tau_2}$ where A1 and A2 are the preexponential-factors of each component.

- Three exponential decay model:

$$\tau_{AVG} = \tau_1 w_1 + \tau_2 w_2 + \tau_3 w_3$$

with weights defined as $w_1 = \frac{A1\tau_1}{A1\tau_1 + A2\tau_2 + A3\tau_3}$, $w_2 = \frac{A2\tau_2}{A1\tau_1 + A2\tau_2 + A3\tau_3}$ and $w_3 = \frac{A3\tau_3}{A1\tau_1 + A2\tau_2 + A3\tau_3}$ where A1, A2 and A3 are the preexponential-factors of each component.

OLED Fabrication and Characterization

Solution-processed OLEDs with **TCzTRZ-DLC** as the emitter were fabricated according to the following procedure. Patterned ITO-coated glass substrates were irradiated with UV-light and treated with O_3 for 30 min. Onto the ITO substrate, PEDOT:PSS (Clevios CH 8000), diluted with ultrapure water in a 1:1 ratio, was spin-coated and subsequently annealed at 150 °C for 10 min in air. Then, PVK in *o*-dichlorobenzene (10 mg mL^{-1}) was spin-coated and annealed at 120 °C for 10 min in air. The emissive layer was also prepared by spin-coating from chlorobenzene solutions (10 mg mL^{-1}) and then annealed at 100 °C for 10 min under vacuum. The remaining layers, mSiTRZ (12 nm), TmPPPyTz (50 nm), Liq (1 nm), and Al (100 nm), were deposited by vacuum evaporation (SE - 4260, ALS Technology, Japan) at $10^{-5} - 10^{-4}$ Pa. Device characterization was carried out using an

absolute EQE measurement setup equipped with an integrating sphere (C9920-12, Hamamatsu Photonics, Japan) and a source meter (Keithley 2400, Japan).

Literature Examples

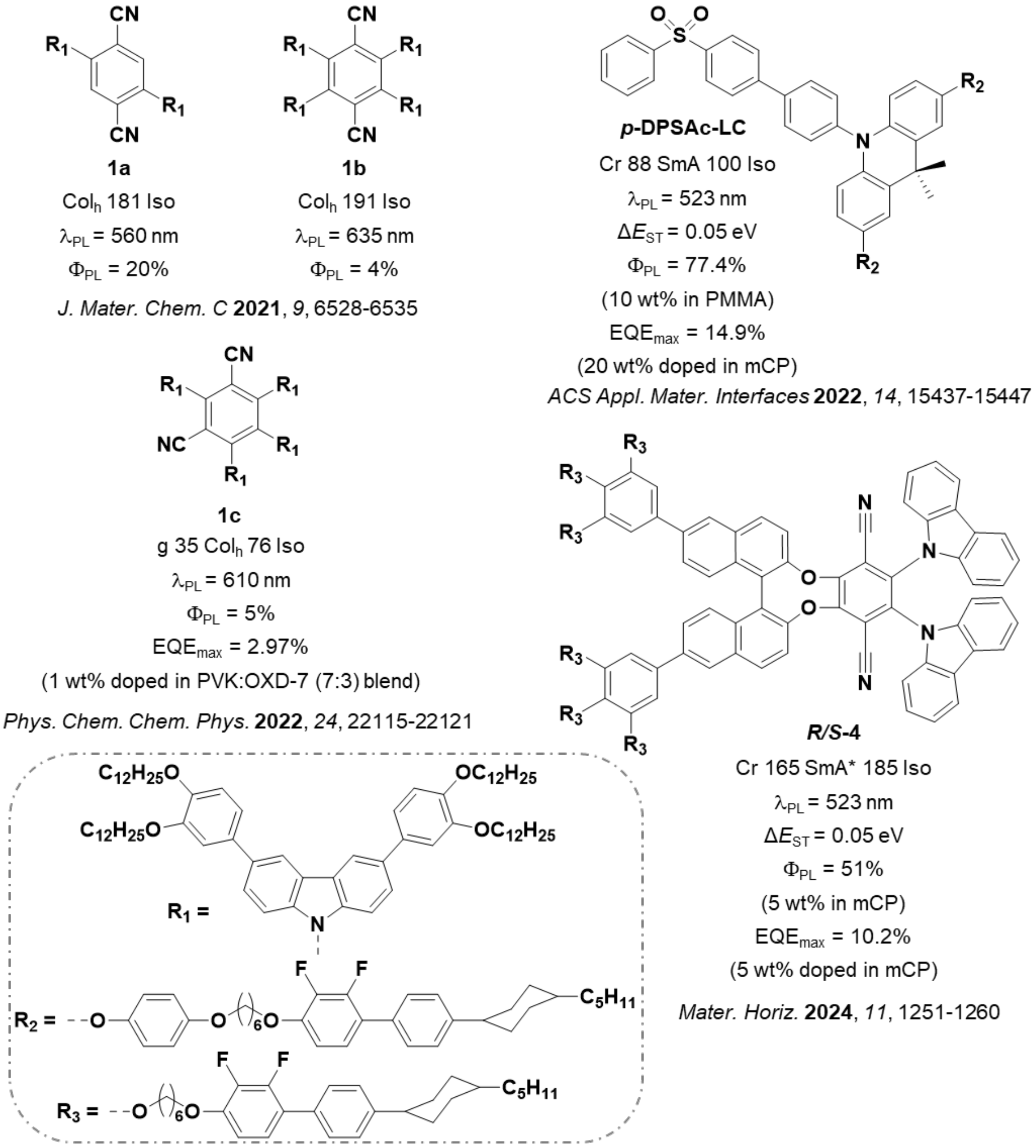

Figure **S1**. Literature survey of liquid crystalline D-A TADF emitters.

Synthetic procedure

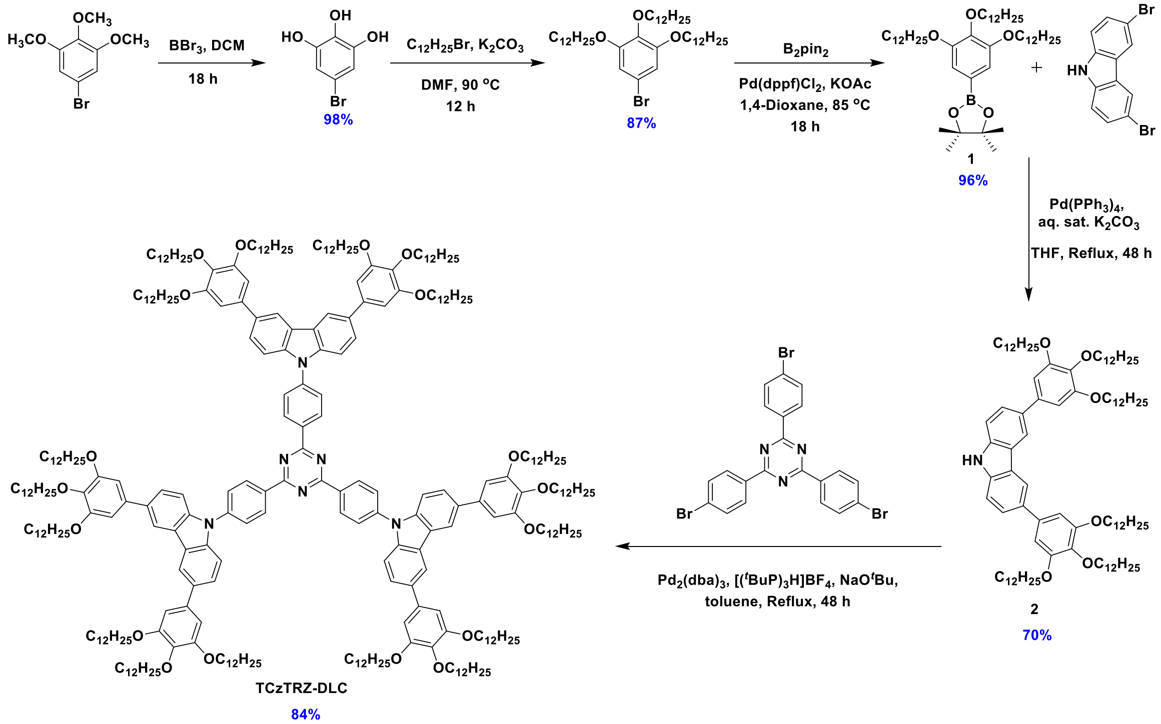

Scheme **S1**. Synthetic routes and chemical structures of **TCzTRZ-DLC**.

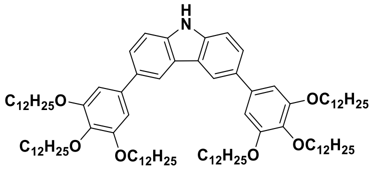

2

Potassium carbonate (704 mg, 5.09 mmol, 4 equiv.), 3,6-dibromo-9H-carbazole (414 mg, 1.27 mmol, 1 equiv.) and 4,4,5,5-tetramethyl-2-(3,4,5-tris(dodecyloxy)phenyl)-1,3,2-dioxaborolane (**1**) (2.12 g, 2.80 mmol, 2.2 equiv.) were added to a mixture of tetrahydrofuran (50 mL) and water (2.5 mL). The resulting solution was degassed with N_2 bubbling for 10 min. $Pd(PPh_3)_4$, (118 mg, 101.8 μmol, 0.08 equiv.) was added and the resulting solution was heated to 75 °C for 48 hours. The reaction was cooled and then tetrahydrofuran was evaporated under reduced pressure. The reaction mixture was extracted with DCM (3 × 100 mL), dried over Na_2SO_4, filtered and concentrated under reduced pressure to give an orange oil. The product was purified by column chromatography on silica gel (2.5% EtOAc:Hexanes). The corresponding fractions were combined and concentrated under reduced pressure to afford a white sticky liquid.

Yield: 70 %. **R*f*:** 0.74 (10% EtOAC:Hexane on silica gel). **^{1}H NMR (400 MHz, $CDCl_3$) δ (ppm):** 8.26 (s, 2H), 8.10 (s, 1H), 7.62 (d, *J* = 8.6 Hz, 2H), 7.47 (d, *J* = 8.8 Hz, 2H), 6.87

(s, 4H), 4.09 (t, J = 5.64 Hz, 8H), 4.01 (t, J = 6.44 Hz, 4H), 1.86-1.76 (m, 12H), 1.57-1.49 (m, 12H), 1.36-1.26 (m, 96H), 0.88-0.86 (m, 18H). **^{13}C NMR (100 MHz, CDCl$_3$) δ (ppm):** 153.52, 139.46, 137.66, 137.62, 133.64, 125.74, 124.02, 118.91, 110.94, 106.46, 73.75, 69.51, 32.08, 30.56, 29.94, 29.87, 29.83, 29.70, 29.63, 29.57, 29.53, 26.33, 22.85, 14.28. **HR-MS (MALDI): Calculated [M+H ($C_{96}H_{161}NO_6$)]:** 1425.2357 **Found:** 1425.4752.

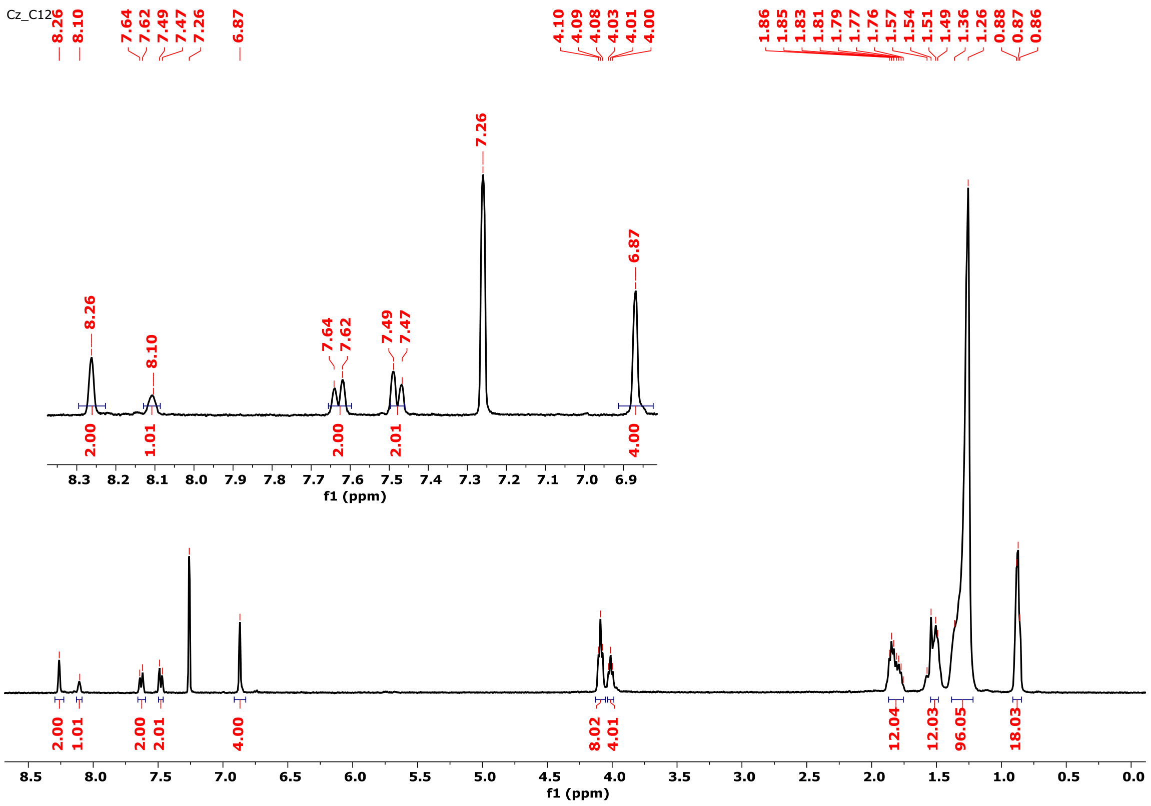

Figure **S2**. ^{1}H NMR spectrum of **2** in CDCl$_3$.

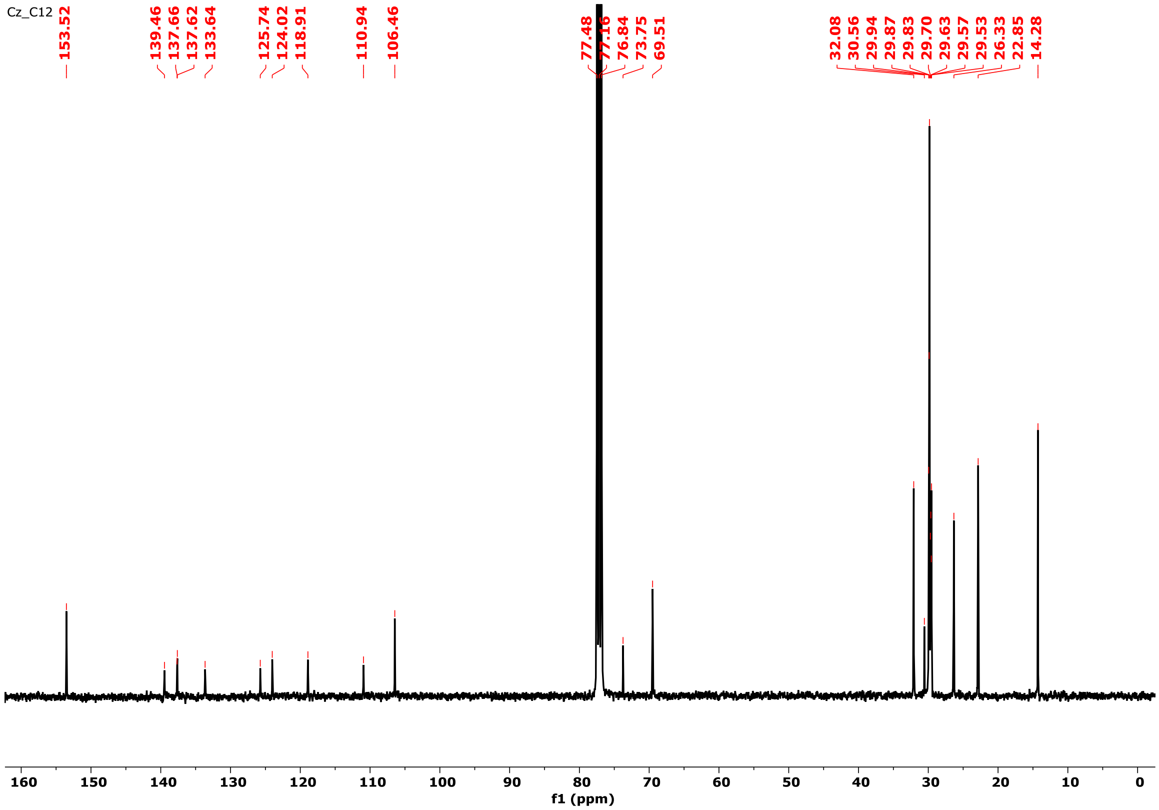

Figure **S3**. ^{13}C NMR spectrum of **2** in $CDCl_3$.

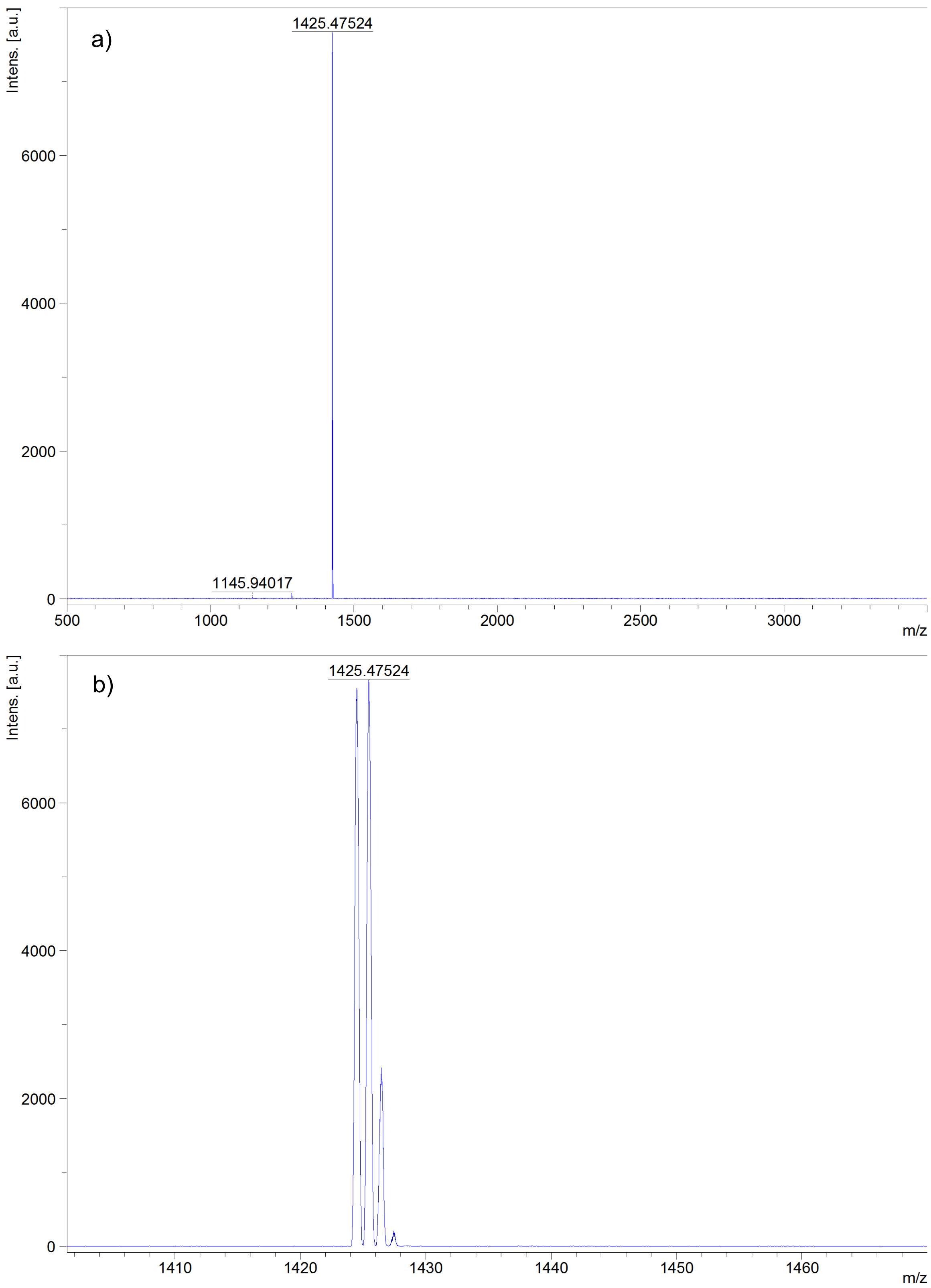

Figure **S4**. MALDI HRMS (a) full spectrum and (b) zoomed spectrum of **2**.

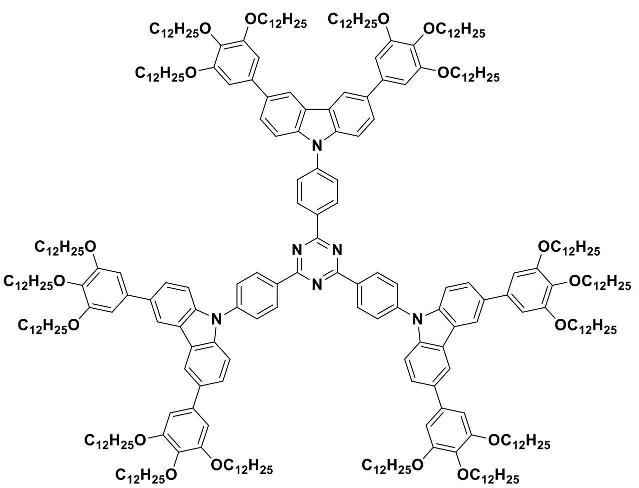

Under a nitrogen atmosphere, 3,6-bis(3,4,5-tris(dodecyloxy)phenyl)-9H-carbazole (0.50 g, 350.8 µmol, 3.3 equiv.) and sodium *tert*-butoxide (0.102 g, 1.063 mmol, 10 equiv.) were dissolved in toluene (8 mL). Tri-*tert*-butylphosphonium tetrafluoroborate (0.0056 g, 19.13 µmol, 0.18 equiv.) was added to the stirring suspension and left to stir for 10 minutes. 2,4,6-tris(4-bromophenyl)-1,3,5-triazine (0.058 g, 106.3 µmol, 1 equiv.) was added and the resulting mixture was stirred for a further 5 minutes, followed by addition of tris(dibenzylideneacetone)dipalladium(0) (0.0088 g, 9.57 µmol, 0.09 equiv.). The solution changed from colourless to dark red immediately. The resulting reaction mixture was left to stir under nitrogen for 48 hours at 120 °C and then concentrated under reduced pressure. The crude mixture was purified by silica gel chromatography using DCM:hexane = 5:95 as eluent to afford the desired product. **Yield**: 84%. **R*f***: 0.7 (hexane : DCM = 2:3 on silica gel). **Mp**: 144-146 °C. **^{1}H NMR (500 MHz, CDCl$_3$) δ (ppm)** 9.18 (d, *J* = 8.65 Hz, 6H), 8.39 (s, 6H), 7.95 (d, *J* = 8.60 Hz, 6H), 7.70-7.66 (m, 12H), 6.93 (s, 12H), 4.14 (t, *J* = 6.45 Hz, 24H), 4.05 (t, *J* = 6.60 Hz, 12H), 1.91-1.80 (m, 36H), 1.56-1.51 (m, 36H), 1.40-1.27 (m, 288H), 0.92-0.87 (m, 54H). **^{13}C NMR (126 MHz, MHz, CDCl$_3$) δ (ppm)** 171.37, 153.60, 142.06, 140.27, 137.85, 137.31, 134.84, 134.78, 131.00, 126.78, 126.02, 124.52, 119.09, 110.31, 106.52, 73.77, 69.57, 32.10, 32.07, 30.56, 29.93, 29.87, 29.82, 29.70, 29.63, 29.56, 26.33, 22.84, 14.27. **HR-MS (MALDI): Calculated ($C_{309}H_{492}N_6O_{18}$):** 4579.79416 **Found:** 4579.29694. **Anal. Calcd. ($C_{309}H_{492}N_6O_{18}$):** C, 81.05; H, 10.83; N, 1.84. **Found:** C, 80.88; H, 10.75; N, 1.74. **HPLC analysis:** 99.52% pure on HPLC analysis, retention time 8.64 minutes in 91% tetrahydrofuran: 9% water mix.

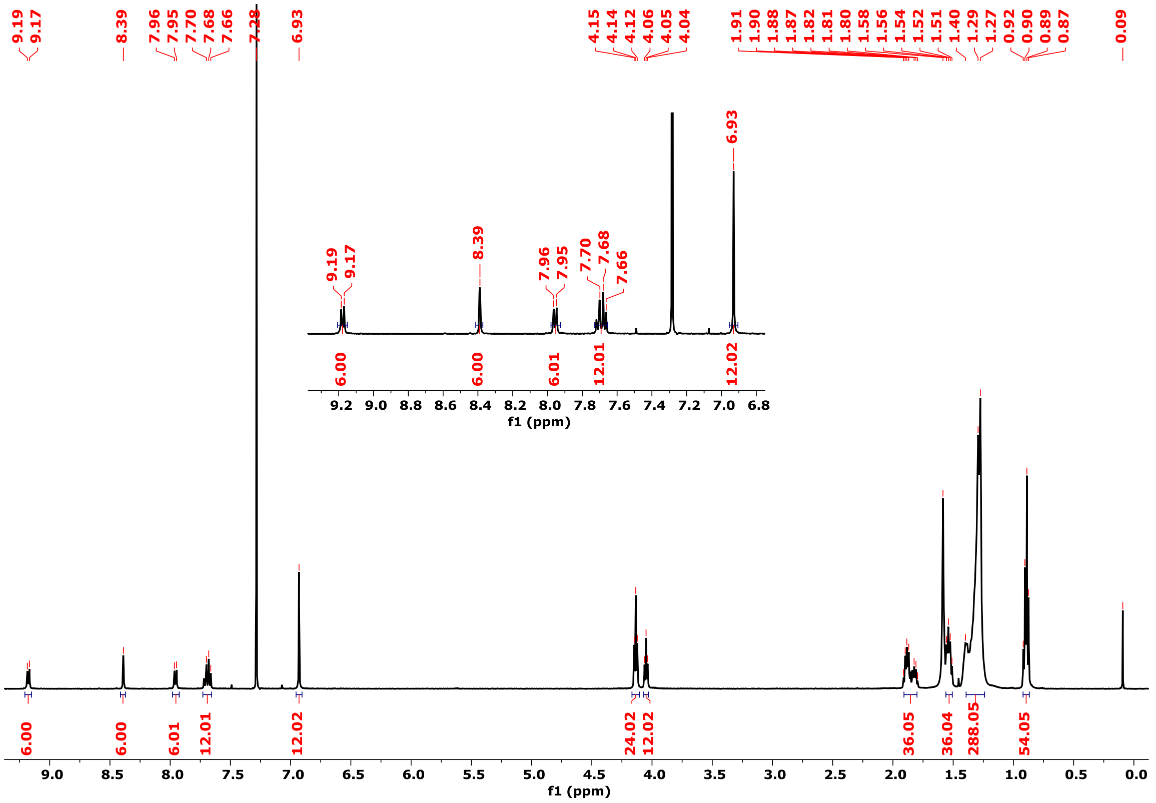

Figure **S5**. ^{1}H NMR spectrum of **TCzTRZ-DLC** in $CDCl_3$.

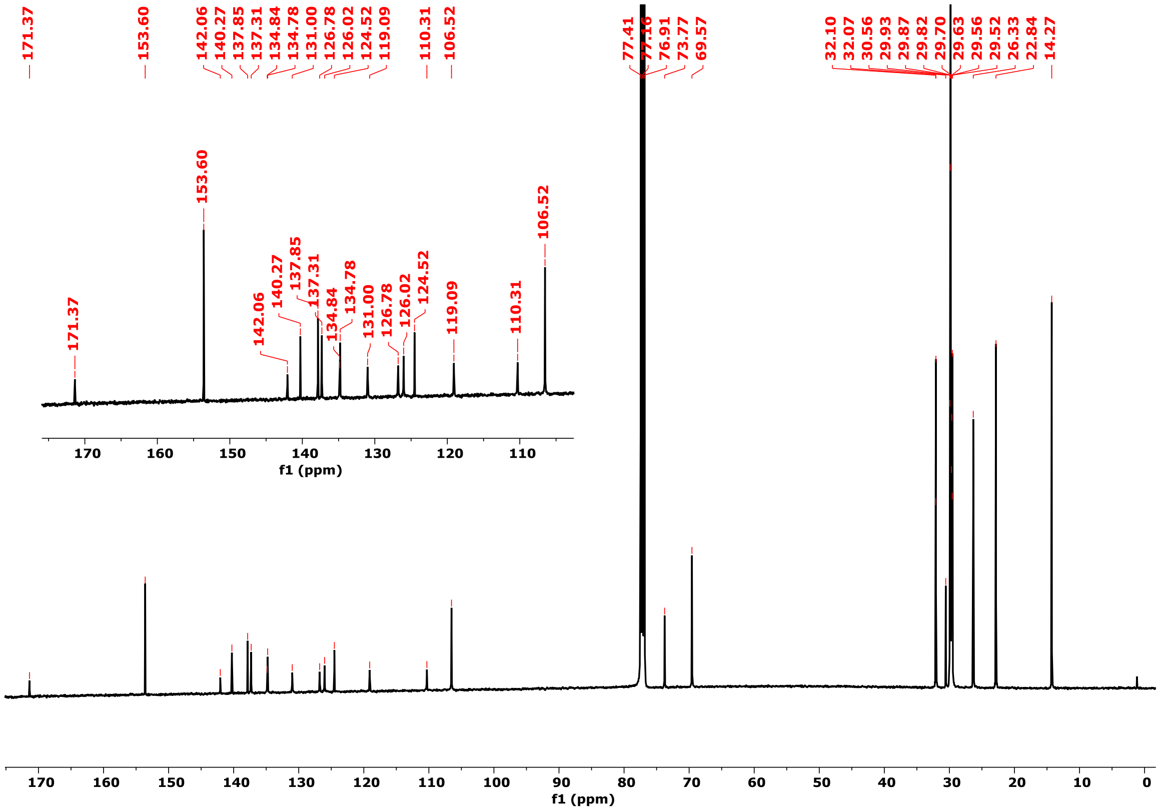

Figure **S6**. ^{13}C NMR spectrum of **TCzTRZ-DLC** in $CDCl_3$.

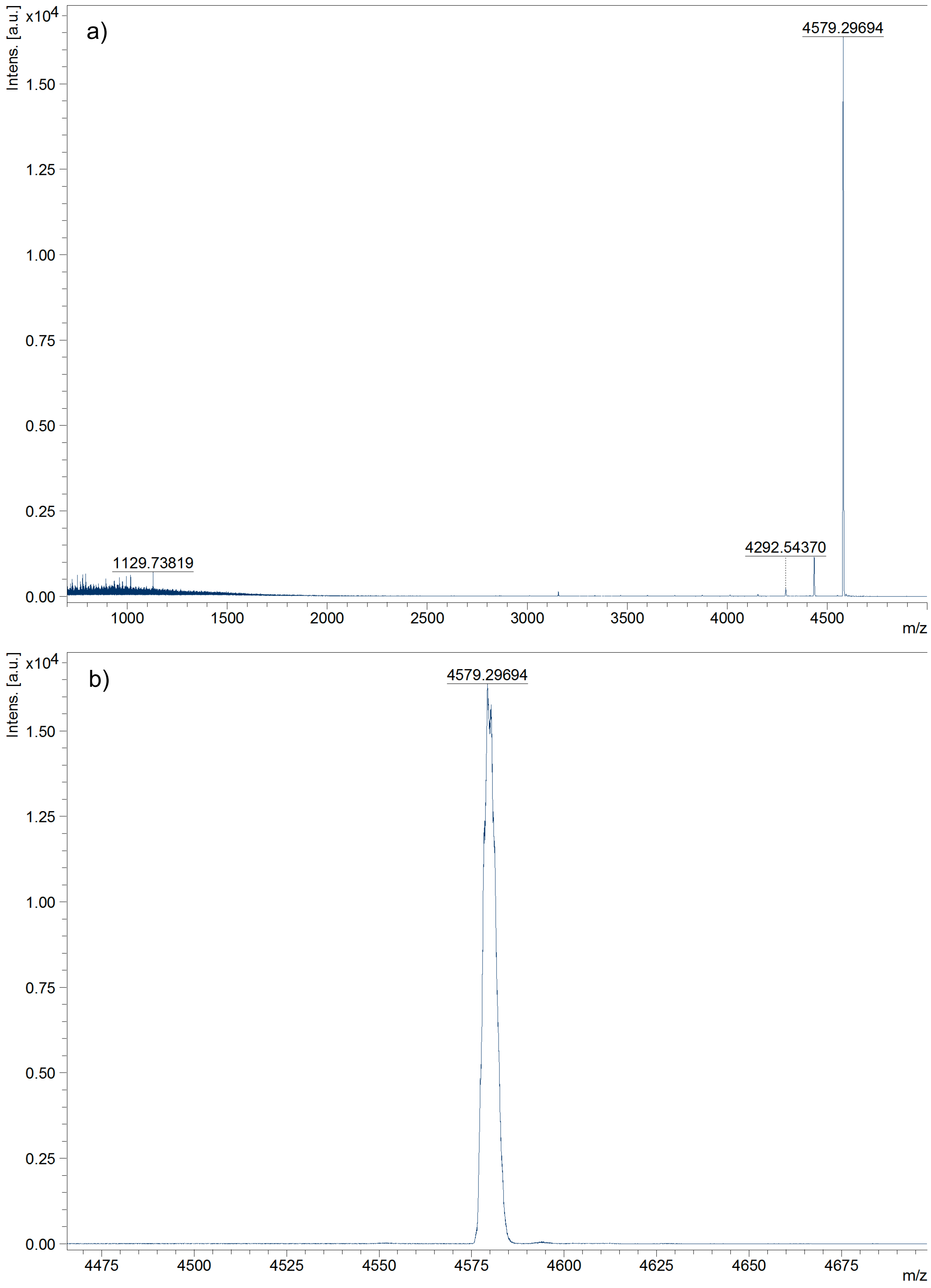

Figure **S7**. MALDI HRMS (a) full spectrum and (b) zoomed spectrum of **TCzTRZ-DLC**.

Elemental Analysis Service Request Form

Researcher name Joydip De

Researcher email jd303@st-andrews.ac.uk

NOTE: Please submit ca. 10 mg of sample

**Sample reference number**	JD-5
**Name of Compound**	TRZ_TCZ_PhC12
**Molecular formula**	C309H492N6O18
**Stability**	
**Hazards**	
**Other Remarks**	

Analysis type:

Single ☐ **Duplicate** ■ **Triplicate** ☐

Analysis Result:

Element	Expected %	Found (1)	Found (2)	Found (3)
Nitrogen	1.84	1.74	1.74	
Carbon	81.05	81.61	80.16	
Hydrogen	10.83	10.86	10.63	

Authorising Signature:

**Date completed**	11·10·23
**Signature**	[illegible]
**comments**	

Figure **S8**. Elemental Analysis of **TCzTRZ-DLC**.

<Sample Information>

Sample Name	: GPC_Fr_3		
Sample ID	:		
Method Filename	: 91% THF 8% water 0.8 mlmin 20 mins.lcm		
Batch Filename	: gpc_fr_repeat_2.lcb		
Vial #	: 1-21	Sample Type	: Unknown
Injection Volume	: 1 uL		
Date Acquired	: 27/08/2023 01:10:20	Acquired by	: System Administrator
Date Processed	: 27/08/2023 01:30:23	Processed by	: System Administrator

<Chromatogram>

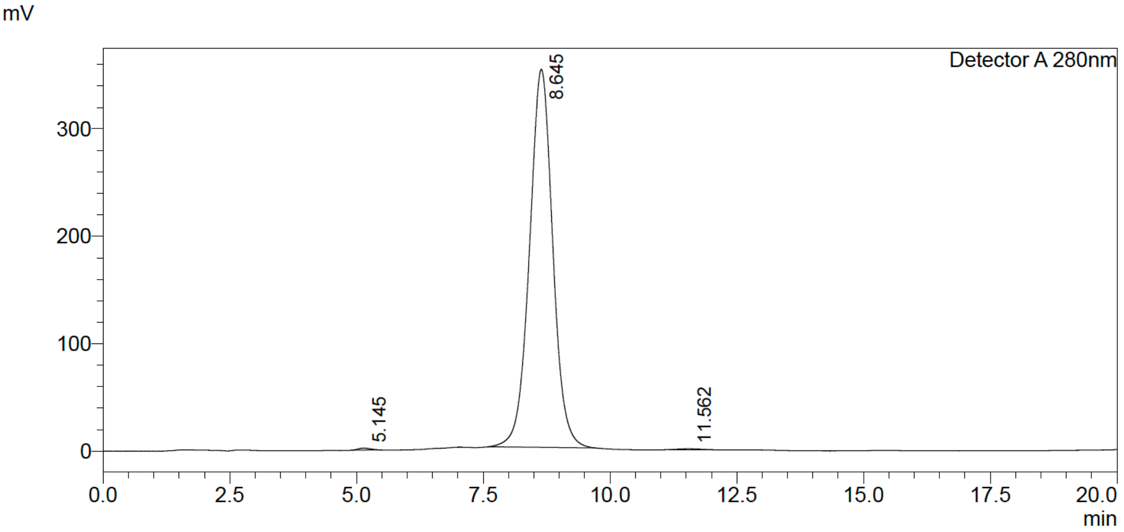

<Peak Table>

Detector A 280nm

Peak#	Ret. Time	Area	Height	Area%	Area/Height	Width at 5% Height
1	5.145	32291	1793	0.286	18.005	0.535
2	8.645	11252790	351974	99.517	31.971	1.115
3	11.562	22310	744	0.197	29.976	0.806
Total		11307392	354511	100.000		

Figure **S9**. HPLC Spectrum of **TCzTRZ-DLC**.

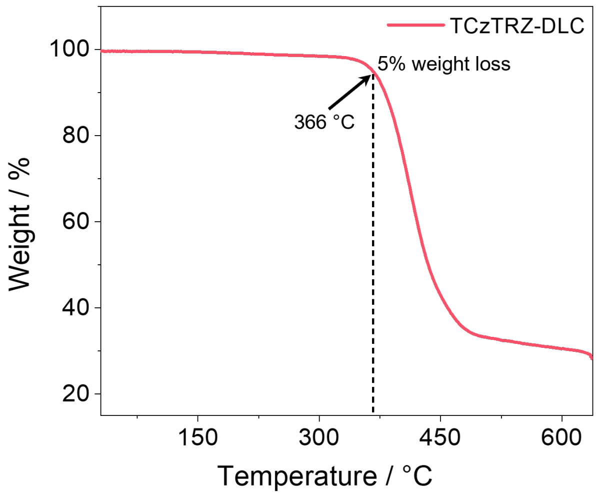

Figure **S10**. TGA curve of **TCzTRZ-DLC**. The measurement was performed under a nitrogen atmosphere, with heating and cooling rates of 10 °C/min.

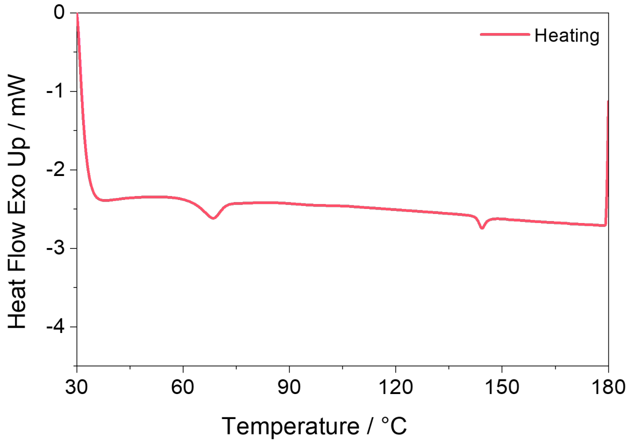

Figure **S11**. DSC thermograms of **TCzTRZ-DLC** upon heating cycle (at 10 °C/min rate).

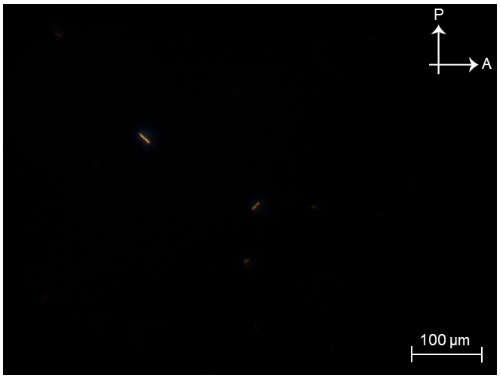

Figure **S12**. POM image of **TCzTRZ-DLC** at 90 °C. Arrows indicate the directions of A: analyser; P: polarizer.

Table **S1**. Experimental data of thermal properties of compound **TCzTRZ-DLC**

**Mesogen**	**Heating Scan**[a] / °C (kJ mol^{-1})	**Cooling Scan**[b] / °C (kJ mol^{-1})
**TCzTRZ-DLC**	Col_h 65.9 (8.84) Col_{sq} 143.1 (1.32) Iso	Iso 141.4 Col_{sq} 68.3 Col_h

[a]Transition temperatures (peak, in °C) and associated enthalpy changes in brackets in kJ mol^{-1} obtained from DSC. [b]Transition temperatures (in °C) obtained from POM. Abbreviations: Col_h = columnar hexagonal, Col_{sq} = columnar square, and Iso = Isotropic liquid.

Table **S2**. Peak assignment of SAXS/WAXS data for compound **TCzTRZ-DLC**

30 °C	1.80 nm^{-1} (100)	3.12 nm^{-1} (110)	3.59 nm^{-1} (200)	4.60 nm^{-1} (210)	14.1 nm^{-1} h_a	17.9 nm^{-1} h_c
90 °C	1.96 nm^{-1} (100)	2.78 nm^{-1} (110)	3.38 nm^{-1} (120)	3.82 nm^{-1} (200)	14.1 nm^{-1} h_a	17.9 nm^{-1} h_c

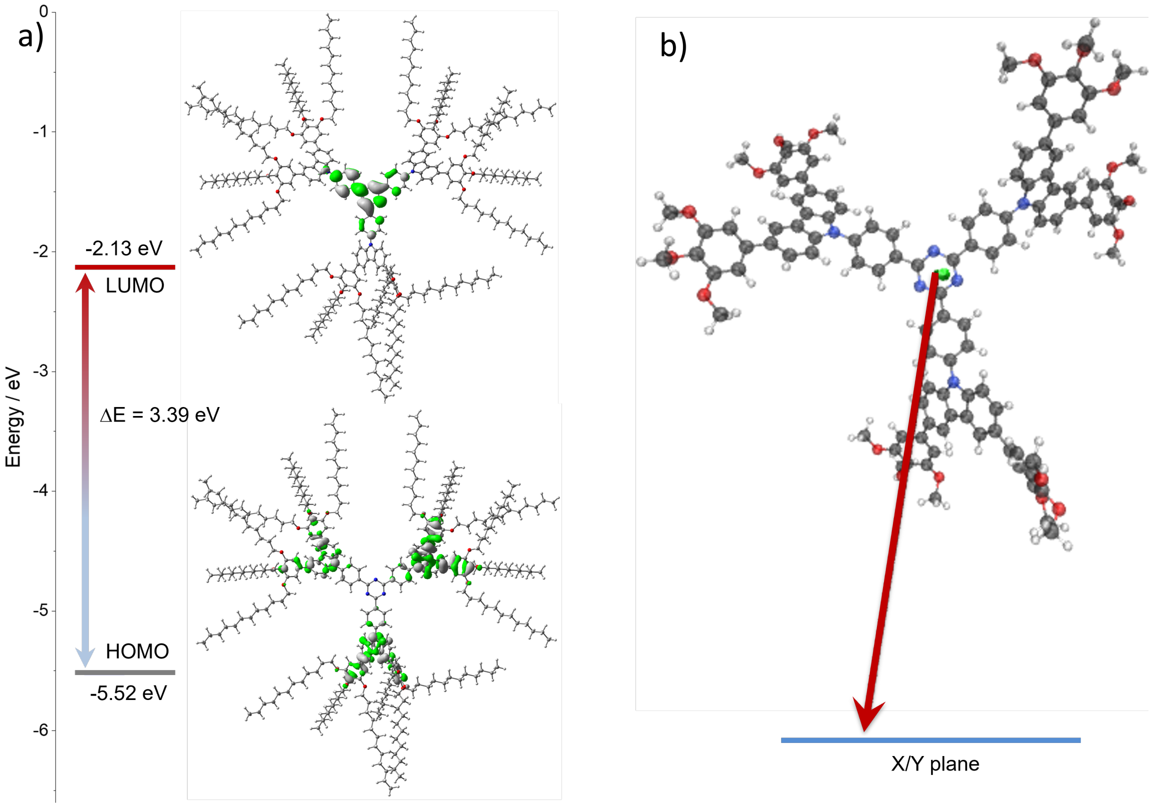

Figure **S13**. (a) Frontier molecular orbitals (isovalue: 0.02) of **TCzTRZ-DLC** calculated in the gas phase at the PEB0/6-31G(d,p) level and (b) TDM vector of **TCzTRZ-OMe** for the S_1 state in the molecular plane (X/Y Plane).

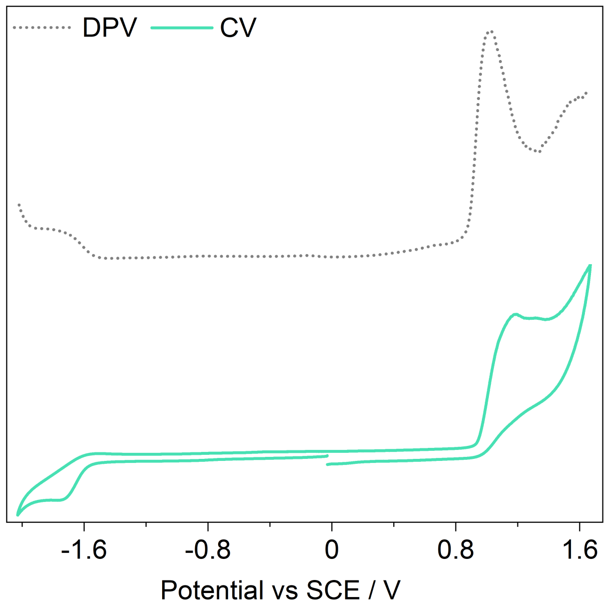

Figure **S14**. Cyclic voltammogram (CV) (solid line) and differential pulse voltammetry (DPV) (dashed line) of **TCzTRZ-DLC** in degassed DCM (scan rate = 100 mV s^{-1}).

Table **S3**. Electrochemical data

Compound	E_{ox} / V[a]	E_{red} / V[a]	ΔE / V[b]	HOMO / eV[c]	LUMO / eV[c]
**TCzTRZ -DLC**	1.02	−1.72	2.74	−5.36	−2.62
**TPT-Cz**[22]	-	-		−6.15[d]	−3.00[e]

[a] E_{ox} and E_{red} are the anodic and cathodic peak potentials, respectively, obtained from DPV using Fc/Fc^+ as the internal reference and referenced *versus* SCE (0.46 V vs. SCE)[3] in degassed DCM with 0.1 M [^{n}Bu$_4$N]PF$_6$ as the supporting electrolyte. [b] $\Delta E = E_{ox} - E_{red}$ [c] $E_{HOMO/LUMO} = -(E_{ox} / E_{red} + 4.8)$ eV,[4] where E_{ox} is the anodic peak potential and E_{red} is the cathodic peak potential calculated from DPV relative to F_c/F_c^+. [d] HOMO value of **TPT-Cz** was determined from ultraviolet photoelectron spectroscopy (UPS), and [e] the LUMO level was estimated by subtracting the optical band gap from the HOMO value, as reported in Ref [22].

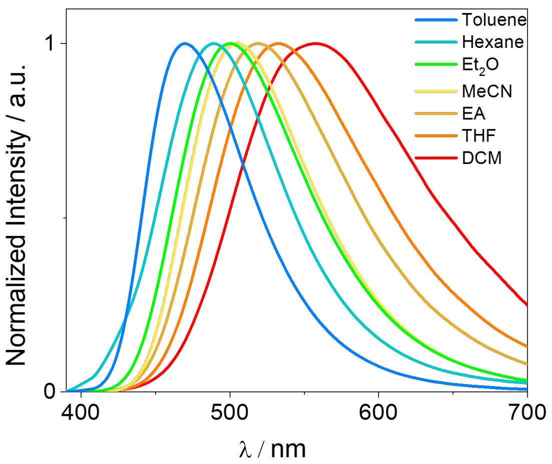

Figure **S15**. Solvatochromatism PL study for **TCzTRZ-DLC** (λ_{exc} = 380 nm).

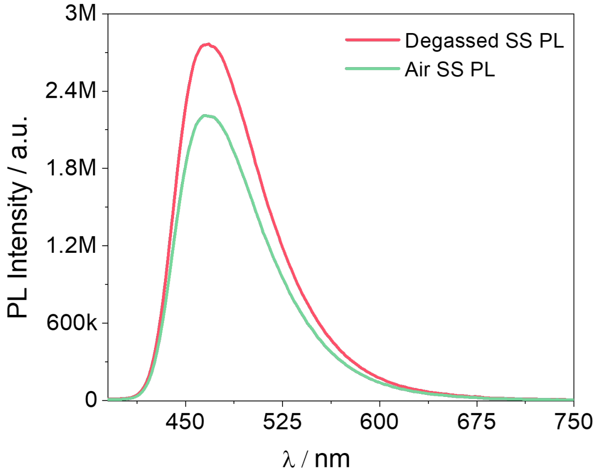

Figure **S16**. Comparison of the steady-state PL of **TCzTRZ-DLC** in both aerated and degassed toluene (λ_{exc} = 380 nm).

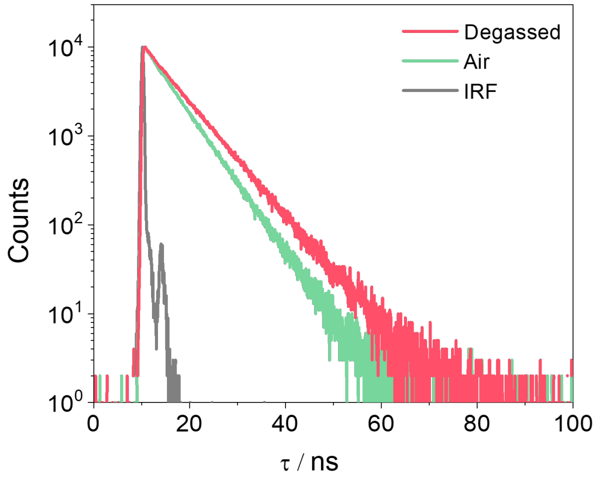

Figure **S17**. Time-resolved PL decay of **TCzTRZ-DLC** in aerated and degassed toluene (λ_{exc} = 379 nm).

Table **S4**. Photoluminescence quantum yield screening in different host matrices and neat film.[a]

		Φ_{PL} / %[b]	
**Emitter**	**N_2**	**Air**	**Film Condition**
**TCzTRZ-DLC**	54.8	46.6	Neat
	12.6	8.1	1 wt% in mCP[c]
	18.4	14.3	3 wt% in mCP[c]
	29.9	21.3	5 wt% in mCP
	71.5	55.3	10 wt% in mCP
	63.7	49.8	20 wt% in mCP
	65.6	51.2	30 wt% in mCP
	56.7	38.6	10 wt% in OXD-7
	81.2	59.1	10 wt% in DPEPO
	59.7	43.3	5 wt% in DPEPO
	53.9	43.4	20 wt% in DPEPO

[a] Φ_{PL} values were determined using an integrating sphere (λ_{exc} = 305 nm); [b] Φ_{PL} values are within an error limit of ± 2%; [c] mCP host emission observed.

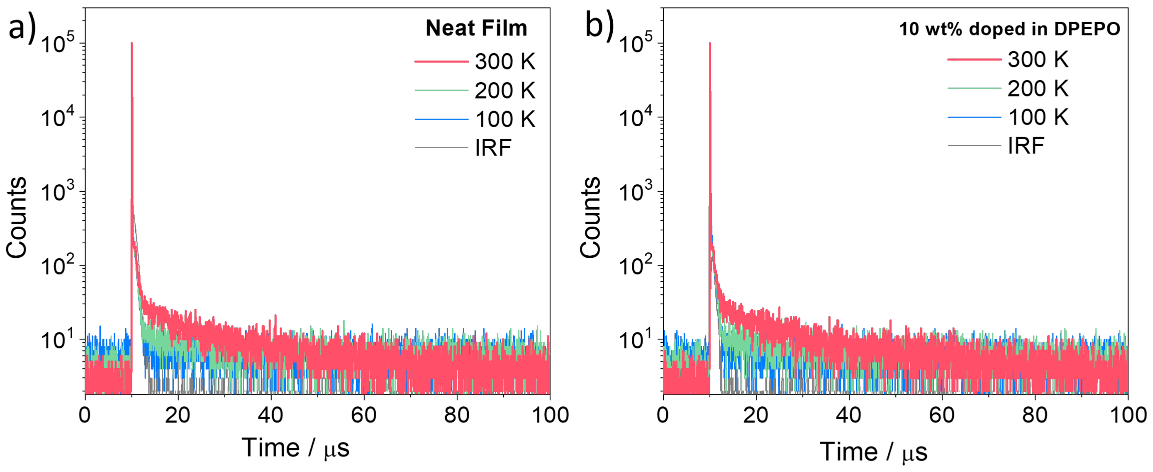

Figure **S18**. Temperature-dependent time-resolved PL decay of **TCzTRZ-DLC** in (a) neat film and (b) DPEPO at 10 wt%, λ_{exc} = 379 nm (Inset shows the Prompt at 300 K).

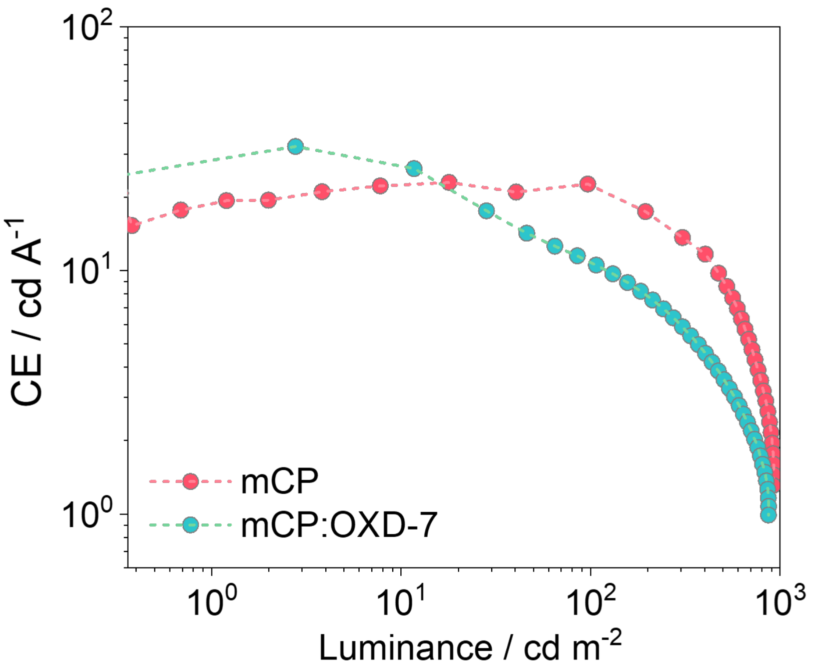

Figure **S19**. Current efficiency *versus* luminance curves of the solution-processed OLEDs with **TCzTRZ-DLC** as D-A DLC-based TADF emitter.

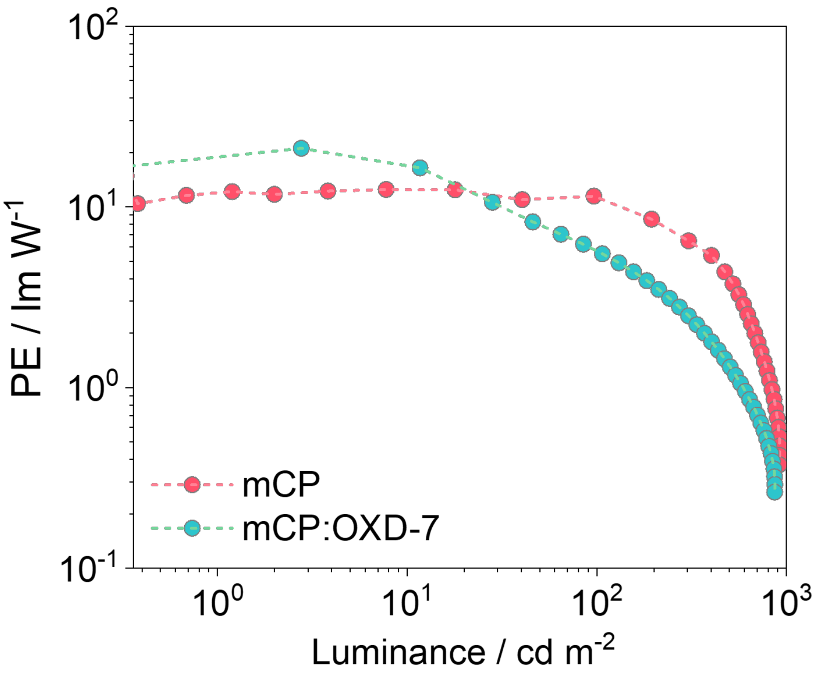

Figure **S20**. Power efficiency *versus* luminance curves of the solution-processed OLEDs with **TCzTRZ-DLC** as D-A DLC-based TADF emitter.

Table **S5**. Data supporting Figure **6f**.

Compound	Class of TADF	Type of Mesophase	Emissive Layer (EML)	λ_{EL} /nm	$EQE_{max/100/1000}$ /%	Ref.
**1a**	D-A	Col_h	1 wt% doped in PVK:OXD-7 (7:3) blend	552	2.29/2.24/-	[23]
**1b**	D-A	Col_h	1 wt% doped in PVK:OXD-7 (7:3) blend	598	0.66/-/-	[23]
**1c**	D-A	Col_h	1 wt% doped in PVK:OXD-7 (7:3) blend	578	2.97/2.85/-	[23]
**TCzTRZ-DLC**	D-A	Col_h	10 wt% doped in mCP	492	9.3/9.2/0.62	**This work**
			10 wt% doped in mCP:OXD-7 (7:3) blend	488	15.5/4.7/0.50	

References:

[1] T. Yasuda, T. Shimizu, F. Liu, G. Ungar, T. Kato, *J. Am. Chem. Soc.* **2011**, *133*, 13437-13444.

[2] G. Zou, L. Zhao, L. Zeng, K. Luo, H. Ni, H. Wang, Q. Li, W. Yu, X. Li, *Inorg. Chem.* **2019**, *58*, 861-869.

[3] N. G. Connelly, W. E. Geiger, *Chem. Rev.* **1996**, *96*, 877-910.

[4] C. M. Cardona, W. Li, A. E. Kaifer, D. Stockdale, G. C. Bazan, *Adv. Mater.* **2011**, *23*, 2367-2371.

[5] M. Frisch, F. J. S. Clemente, V. Barone, B. Mennucci, GA Petersson, H. Nakatsuji, M. Caricato, A. V. Marenich, J. Bloino, B. G. Janesko, R. Gomperts, B. Mennucci, H. P. Hratchian, J. V. Ortiz, A. F. Izmaylov, J. L. Sonnenberg, Williams, F. Ding, F. Lipparini, F. Egidi, J. Goings, B. Peng, A. Petrone, T. Henderson, D. Ranasinghe, V. G. Zakrzewski, J. Gao, N. Rega, G. Zheng, W. Liang, M. Hada, M. Ehara, K. Toyota, R. Fukuda, J. Hasegawa, M. Ishida, T. Nakajima, Y. Honda, O. Kitao, H. Nakai, T. Vreven, K. Throssell, J. A. Montgomery Jr., J. E. Peralta, F. Ogliaro, M. J. Bearpark, J. J. Heyd, E. N. Brothers, K. N. Kudin, V. N. Staroverov, T. A. Keith, R. Kobayashi, J. Normand, K. Raghavachari, A. P. Rendell, J. C. Burant, S. S. Iyengar, J. Tomasi, M. Cossi, J. M. Millam, M. Klene, C. Adamo, R. Cammi, J. W. Ochterski, R. L. Martin, K. Morokuma, O. Farkas, J. B. Foresman, D. J. Fox, *Gaussian 16 Rev. C.01*, Wallingford, CT, 2016.

[6] C. Adamo, V. Barone, *J. Chem. Phys.* **1999**, *110*, 6158-6170.

[7] T. H. Dunning, Jr., *J. Chem. Phys.* **1989**, *90*, 1007-1023.

[8] S. Grimme, *Chem. Phys. Lett.* **1996**, *259*, 128-137.

[9] S. Hirata, M. Head-Gordon, *Chem. Phys. Lett.* **1999**, *314*, 291-299.

[10] O. S. Lee, E. Zysman-Colman, *Digichem (version 7) Chemicus Limited*, St Andrews, Scotland, **2025**.

[11] O. S. Lee, M. C. Gather, E. Zysman-Colman, *Digital Discovery* **2024**, *3*, 1695-1713.

[12] N. M. O'Boyle, A. L. Tenderholt, K. M. Langner, *J. Comput. Chem.* **2008**, *29*, 839-845.

[13] W. Humphrey, A. Dalke, K. Schulten, *J. Mol. Graph.* **1996**, *14*, 33-38.

[14] J. E. Stone, *Computer Science Department, University of Missouri-Rolla,* **1998**.

[15] J. D. Hunter, *Comput. Sci. Eng.* **2007**, *9*, 90-95.

[16] N. M. O'Boyle, M. Banck, C. A. James, C. Morley, T. Vandermeersch, G. R. Hutchison, *J. Cheminform.* **2011**, *3*, 33.

[17] N. M. O'Boyle, C. Morley, G. R. Hutchison, *Chem. Cent. J.* **2008**, *2*, 5.

[18] X. Gao, S. Bai, D. Fazzi, T. Niehaus, M. Barbatti, W. Thiel, *J. Chem. Theory Comput.* **2017**, *13*, 515-524.

[19] G. A. Crosby, J. N. J. T. J. o. P. C. Demas, **1971**, *75*, 991-1024.

[20] W. H. J. T. J. o. P. C. Melhuish, **1961**, *65*, 229-235.

[21] N. C. Greenham, I. D. W. Samuel, G. R. Hayes, R. T. Phillips, Y. A. R. R. Kessener, S. C. Moratti, A. B. Holmes, R. H. Friend, *Chem. Phys. Lett.* **1995**, *241*, 89-96.

[22] J. Xiao, X.-K. Liu, X.-X. Wang, C.-J. Zheng, F. Li, *Org. Electron.* **2014**, *15*, 2763-2768.

[23] A. F. Suleymanova, M. Z. Shafikov, X. Chen, Y. Wang, R. Czerwieniec, D. W. Bruce, *Phys. Chem. Chem. Phys.* **2022**, *24*, 22115-22121
